# Genotoxic stress stimulates eDNA release *via* explosive cell lysis and thereby promotes streamer formation of *Burkholderia cenocepacia* H111 cultured in a microfluidic device

**DOI:** 10.1038/s41522-023-00464-7

**Published:** 2023-12-09

**Authors:** Zaira Heredia-Ponce, Eleonora Secchi, Masanori Toyofuku, Gabriela Marinova, Giovanni Savorana, Leo Eberl

**Affiliations:** 1 Department of Plant and Microbial Biology, University of Zürich, 8008 Zürich, Switzerland; 2 Institute of Environmental Engineering, Department of Civil, Environmental and Geomatic Engineering, ETH Zürich, 8093 Zürich, Switzerland; 3 Faculty of Life and Environmental Sciences, Microbiology Research Center for Sustainability (MiCS), University of Tsukuba, 1-1-1 Tennodai, Tsukuba, Ibaraki Japan

**Keywords:** Biofilms, Pathogens

## Abstract

DNA is a component of biofilms, but the triggers of DNA release during biofilm formation and how DNA contributes to biofilm development are poorly investigated. One key mechanism involved in DNA release is explosive cell lysis, which is a consequence of prophage induction. In this article, the role of explosive cell lysis in biofilm formation was investigated in the opportunistic human pathogen *Burkholderia cenocepacia* H111 (H111). Biofilm streamers, flow-suspended biofilm filaments, were used as a biofilm model in this study, as DNA is an essential component of their matrix. H111 contains three prophages on chromosome 1 of its genome, and the involvement of each prophage in causing explosive cell lysis of the host and subsequent DNA and membrane vesicle (MV) release, as well as their contribution to streamer formation, were studied in the presence and absence of genotoxic stress. The results show that two of the three prophages of H111 encode functional lytic prophages that can be induced by genotoxic stress and their activation causes DNA and MVs release by explosive cell lysis. Furthermore, it is shown that the released DNA enables the strain to develop biofilm streamers, and streamer formation can be enhanced by genotoxic stress. Overall, this study demonstrates the involvement of prophages in streamer formation and uncovers an often-overlooked problem with the use of antibiotics that trigger the bacterial SOS response for the treatment of bacterial infections.

## Introduction

Biofilms are bacterial communities enclosed in an extracellular matrix of polysaccharides, proteins, nucleic acids, and lipids^1^. The matrix components interact with each other, such as polysaccharides with eDNA (extracellular DNA) and polysaccharides with proteins, to form an interconnected mesh that surrounds the cells and protects them against environmental stressors^2–6^. The importance of DNA in biofilm formation was reported for the first time just twenty years ago in a study made on *Pseudomonas aeruginosa*^7^. Since then, DNA has been shown to be important for biofilm formation in many other bacterial species. Previous studies have shown that DNA-degrading enzymes (DNases) can inhibit biofilm formation, disperse preformed biofilms or weaken their structure in *P. aeruginosa*, *Listeria monocytogenes*, *Vibrio cholerae* and *Staphylococcus aureus*^8–12^. eDNA has also been shown to be an essential component of streamers, which are flow-suspended biofilm filaments^13,14^. Although the importance of DNA to maintain and modulate biofilm structure is unquestionable, the triggers of DNA release during biofilm formation and the integration of DNA into the biofilm matrix are poorly understood. Several studies have demonstrated that the DNA in the biofilm matrix originates from the genomic DNA of bacteria within the biofilm^15–17^. According to the literature, DNA can be released from bacterial cells into the extracellular space through active secretion, cell lysis or via MVs^18,19^. Cell lysis can occur either from autolysis, or prophage induction^20^. It has been previously reported that autolysis plays an essential role in DNA release and biofilm formation in the Gram-positive bacteria *S. aureus, Staphylococcus epidermidis*, and *Enterococcus faecalis*. In these species, the deletion of autolysin genes (*cidA, atlE*, and *atn*, respectively) reduced the amount of DNA in biofilms and had a dramatic impact on biofilm morphology and adherence^15,21–23^. Prophage induction causes host cell lysis and, consequently, DNA release in subpopulations of *Streptococcus pneumoniae* and *Shewanella oneidensis*, leading to enhanced biofilm formation^24,25^. In interstitial biofilms of *P. aeruginosa*, cell lysis triggered by a phage-derived endolysin encoded in the bacterial genome causes DNA and membrane vesicles (MVs) release^26^. Since prophage induction causes DNA release^27^ and DNA forms the backbone of streamers^14^, we speculate that prophage induction might control streamer formation.

Prophages are common in prokaryotic genomes, especially in pathogens, where they may account for as much as 20% of their genome^28–31^. Temperate prophages remain stably integrated into the host genome and replicate along with the host. However, they can enter the lytic cycle in response to certain environmental conditions, typically genotoxic stress, such as exposure to UV light or DNA-damaging agents^27,32,33^. Genotoxic stress activates the bacterial SOS response, a conserved and widespread general stress response system that allows bacteria to respond to DNA damage. Regulation *via* the SOS system depends on two regulatory proteins^34^: the LexA repressor, which inhibits expression of SOS genes during regular growth by blocking their transcription through binding to specific DNA sequences (SOS boxes) in the promoter regions of target genes^35^ and the RecA protein, which binds to single-stranded DNA (ssDNA) generated by DNA damage or inhibition of replication. The SOS response is activated after DNA damage by the accumulation of ssDNA, around which RecA forms a filament and becomes activated^36^. The activated form of RecA interacts with the LexA repressor and facilitates its self-cleavage^37^. As a consequence, expression of SOS genes is de-repressed. In most bacteria, the SOS regulon comprises genes required for different DNA repair and recombination pathways^38,39^, cell division and septum formation^40^. Importantly, RecA also activates self-cleavage of phage repressors and thus induces the expression of lytic genes that promote DNA replication, phage particle assembly, DNA packaging, host DNA degradation and eventually bacterial lysis^41,42^. The most commonly used agent to induce the SOS response is mitomycin C (MMC), which forms covalent bonds between DNA strands and thereby prevents DNA replication and transcription^43^. Although MMC shows antibacterial activity, it is primarily used as an anticancer drug in chemotherapy to treat various types of cancer in the clinic^44^. Some other antibiotics, particularly quinolones such as ciprofloxacin (CPFLX), which inhibits the bacterial DNA gyrase and topoisomerase IV, also induce the cellular SOS response by blocking DNA synthesis and replication^45^. CPFLX is primarily used as an antibiotic to treat a wide variety of bacterial infections, including lung infections in cystic fibrosis patients (CF)^46^.

In this study, we investigated the role of explosive cell lysis in streamer formation in the opportunistic human pathogen *B. cenocepacia* H111. Representatives of *B. cenocepacia* are problematic opportunistic pathogens in patients suffering from CF, structural lung diseases and in immunocompromised individuals^47–49^. In particular, H111 was isolated from the sputum sample of a cystic fibrosis patient and carries three independent prophages in its genome^50,51^. In this study, we evaluated the contribution of each of these prophages to eDNA release and streamer formation of H111 in the absence and presence of genotoxic stress by using the DNA-damaging agents MMC and CPFLX.

## Results

### *B. cenocepacia* H111 carries three putative prophages in its genome

Prophages are viruses whose genetic material has been incorporated into the bacterial genome or exist as an extrachromosomal plasmid within a bacterial host cell^31^. *B. cenocepacia* H111 has three prophages in chromosome 1 of its genome^51^. These prophages were previously identified by the prophage-finding program PHAge Search Tool (PHAST), and one of them was affiliated with the *myovirus* family^51^. In full agreement with this study, we identified the same three putative prophage regions in the *B. cenocepacia* H111 genome using an updated version of the PHAST program (PHASTER)^52,53^. The previously characterized phage of H111, which corresponds to the phage encoded in region 3 (Supplementary Tables 1 and 2), shows a relatively narrow infection range outside *B. cenocepacia*^51^. Updated information on the three phage regions, including the length of prophage DNA, their exact location in the genome, the number of phage proteins and annotations can be found in Supplementary Tables 1 and 2.

### Genotoxic stress induces prophages in *B. cenocepacia* H111

**Fig. 1:**
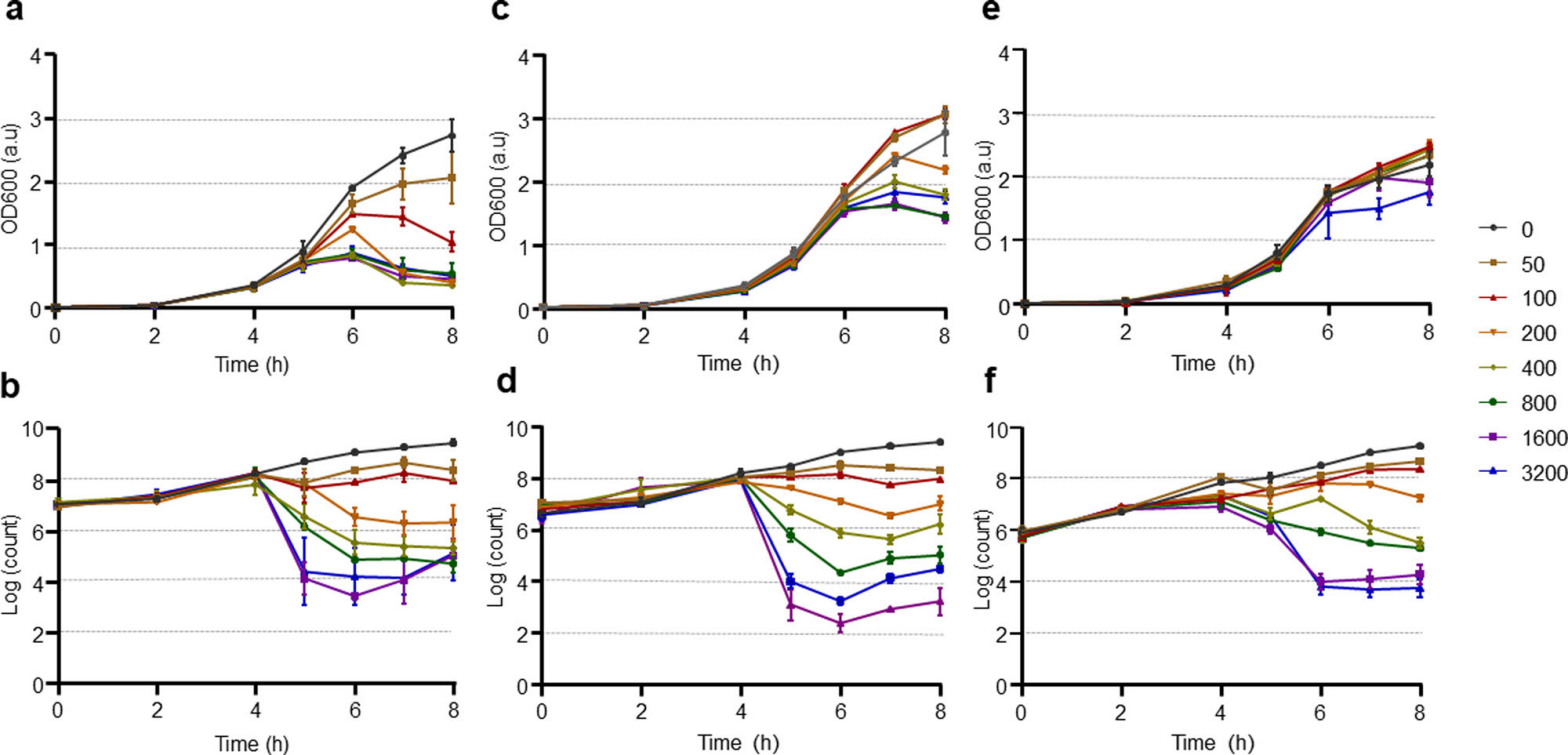
The effect of MMC on growth of B. cenocepacia H111. **a**, **b** Growth curves of *B. cenocepacia* H111 wildtype in the presence of different MMC concentrations (**a** OD600; **b** log CFU mL^−1^); **c**, **d** Growth curves of *B. cenocepacia* H111 *Δlys1,lys2,lys3* in the presence of different MMC concentrations (**c** OD600; **d** log CFU mL^−1^); **e**, **f** growth curves of *P. putida* IsoF in the presence of different MMC concentrations (**e** OD600; **f** log CFU mL^−1^). Error bars represent the standard error of the mean (SEM). Charts show data obtained from three independent experiments. MMC concentrations are expressed in ng/mL^−1^.

The transition between lysogenic and lytic cycles can occur spontaneously at a low frequency, but is often strongly increased by genotoxic stress^33,54^. To investigate whether prophage induction in *B. cenocepacia* H111 is influenced by genotoxic stress, growth of the H111 wildtype strain and the H111 *Δlys1,lys2,lys3* triple mutant, in which all the three putative endolysins identified in the H111 prophage regions were deleted, was followed by determining CFUs/ml and measuring optical density at 600 nm. As a control, we also included *P. putida* IsoF (IsoF), a strain that naturally does not have a prophage in its genome. CFUs decreased with higher MMC concentrations in all three strains tested. However, optical density measurements were more similar between control and treatments in both H111 *Δlys1,lys2,lys3* and IsoF compared to the H111 wildtype (Fig. 1), suggesting increased resistance to cell lysis in the absence of functional endolysins. MMC 200 ng mL^−1^ was chosen for further experiments, as in our experimental conditions this concentration induces prophages but does not kill the entire bacterial population (Fig. 1).

### Prophage induction causes DNA and MVs release from *B. cenocepacia* H111

**Fig. 2:**
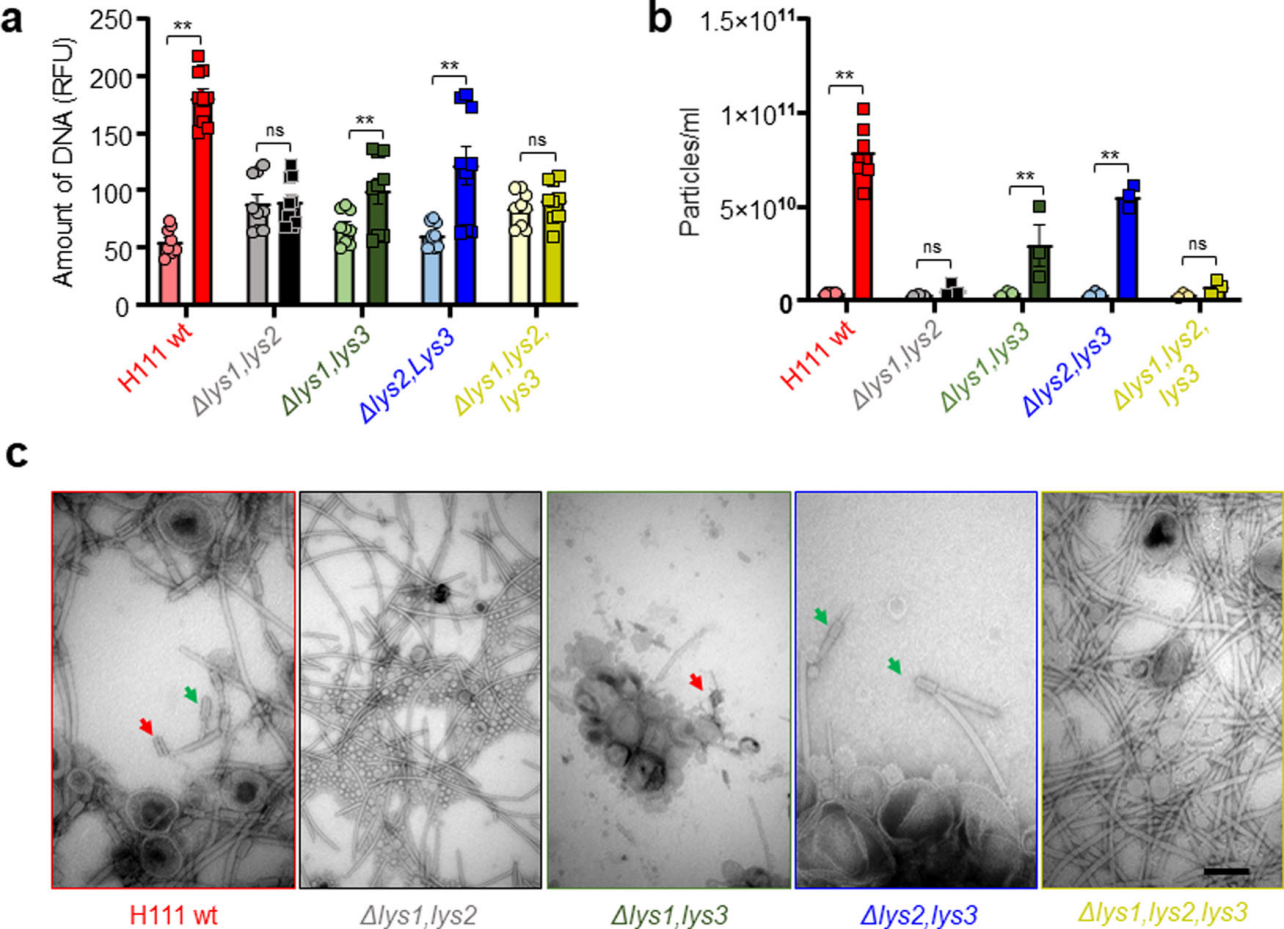
The role of prophage-encoded endolysins in MMC-triggered cell lysis. **a** Amount of DNA (SYTOX fluorescence) in cell-free supernatants of the H111 wildtype and its derived endolysin mutant strains grown without treatment (left, circles) and treated with MMC (right, squares). Individual measurements are represented with geometrical shapes and the mean is represented with bars. Three biological replicates, each with three technical replicates, were performed. **b** Amount of membrane vesicles (particles/ml) in cell-free supernatants of the H111 wildtype and its derived endolysin mutant strains grown without treatment (left, circles) and treated with MMC (right, squares). Individual measurements are represented with geometrical shapes and the mean is represented with bars. From three to six biological replicates were performed. Each individual measurement (geometrical shape) represents the average value of 15 technical replicates. **c** Representative TEM images of membrane vesicles extractions from cultures of the H111 wildtype and its derived endolysin mutant strains, all treated with MMC. Different phage structures are indicated with arrows in different colours (red, green). Error bars represent the standard error of the mean (SEM); scale bar 100 nm. Statistical significance between control and treatment was determined using the Holm–Sidak method, with *α* = 0.05; *, *P* < 0.05; **, *P* < 0.01; ns not significant, RFU relative fluorescence units.

The influence of genotoxic stress in prophage induction and subsequent cell lysis was further investigated by determining the amounts of extracellular DNA (eDNA) and MVs in cell-free supernatants of untreated and MMC-treated cultures of the H111 wildtype strain, three double endolysin mutants (H111 *Δlys1,lys2*, H111 *Δlys1,lys3* and H111 *Δlys2,lys3*) and the triple endolysin mutant H111 *Δlys1,lys2,lys3*. The results show that the H111 wildtype releases about two to three times more DNA to the extracellular space when treated with MMC when compared to its control (Fig. 2a). MMC treatment of the H111 wildtype caused DNA release via explosive cell lysis, as observed by epifluorescence microscopy (Video 1). The amount of DNA released by the double mutants H111 *Δlys1,lys3*, and H111 *Δlys2,lys3* increased by a factor of about 2 upon MMC treatment, while no significant increase was observed in the H111 *Δlys1,lys2* and the H111 *Δlys1,lys2,lys3* strains (Fig. 2a). In addition to eDNA, the H111 wildtype, H111 *Δlys1,lys3*, and H111 *Δlys2,lys3* released substantially more MVs to the extracellular space when treated with MMC relative to the untreated controls (Fig. 2b). As with eDNA release, this was not the case with strains H111 *Δlys1,lys2* and H111 *Δlys1,lys2,lys3* (Fig. 2a, b). Likewise, increased eDNA release into the surrounding medium was observed when CPFLX, a fluoroquinolone antibiotic that also induces the SOS response, was used instead of MMC (Supplementary Fig. 1). It is important to note that all strains used in these experiments produced MVs with a similar size distribution regardless of whether they had been treated with MMC or not (Supplementary Fig. 2). Using TEM, two morphologically different phage structures were observed in MVs preparations of the H111 wildtype, while only one of the two was detected in samples of the double mutants H111 *Δlys1,lys3* and in H111 *Δlys2,lys3* (Fig. 2c). The MVs preparations of the H111 *Δlys1,lys3* strain showed phage structures that resemble tail sheaths, and MVs preparations of the H111 *Δlys2,lys3* include contractile phage tail structures with tail fibres (Fig. 2c). No phage structures were observed in MVs samples of *Δlys1,lys2* and H111 *Δlys1,lys2,lys3* strains (Fig. 2c). Collectively, these results suggest that prophage regions containing *lys1* and *lys2* endolysins encode phage tails that, when induced, lyse the host cell. By contrast, the prophage region containing *lys3* appears to be compromised and is not involved in the SOS-induced cell lysis.

**Fig. 3:**
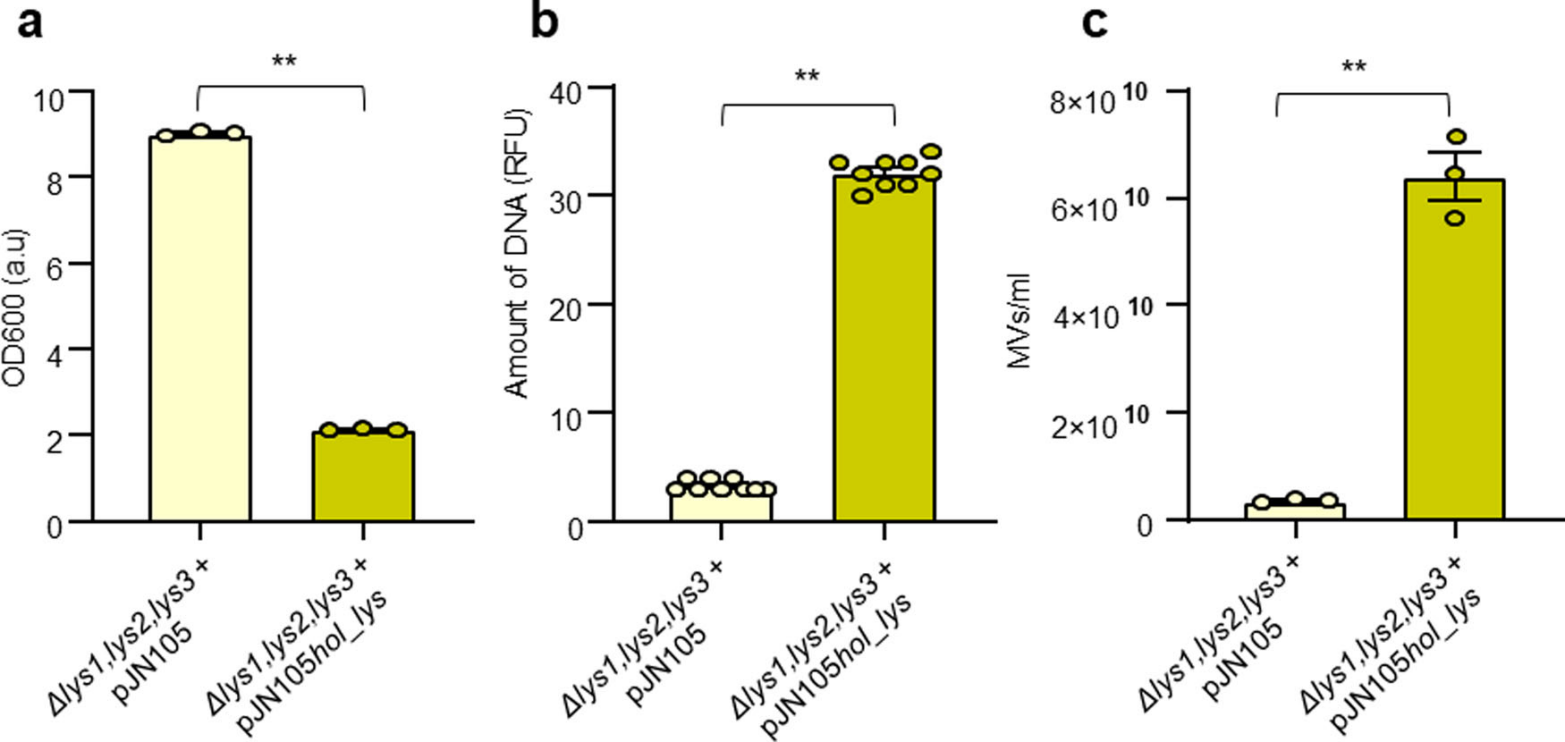
eDNA release and MV formation depends on endolysin-triggered explosive cell lysis in *B. cenocepacia* H111. Heterologous expression of an endolysin-holin cassette of *P. aeruginosa* PAO1 in the H111 *Δlys1,lys2,lys3* mutant strain caused explosive cells lysis and concomitant release of eDNA and MVs. The H111 *Δlys1,lys2,lys3* mutant carrying the empty pJN105 plasmid was used as a control. Cultures were grown for 4 h prior to the induction of the endolysin–holin cassette by the addition of 2% arabinose. Samples were taken after 20 h of growth under inducing conditions. **a** OD600; **b** amount of eDNA in cell-free culture supernatants as determined by staining with SYTOX; **c** amount of membrane vesicles (particles/ml) in cell-free culture supernatants. Charts show data obtained from three independent biological replicates. Error bars represent the standard error of the mean (SEM). Statistical significance was calculated using an unpaired *t*-test with *α* = 0.05; *P < 0.05; **P < 0.01; RFU relative fluorescence units.

To evaluate whether MMC or CPFLX treatments could cause eDNA release as a consequence of bacterial killing by the antibiotic rather than by triggering explosive cell lysis due genotoxic stress, we decided to heterologously express an holin-endolysin cassette of *P. aeruginosa* PAO1, in the H111 *Δlys1,lys2,lys3* mutant strain (Fig. 3). The construct used contains the genes PA0614 and PA0629 encoding a holin and the endolysin Lys^26^, respectively, under the control of an arabinose-inducible promoter. The holin, which generate pores in the cytoplasmic membrane, is required for the endolysin to reach the peptidoglycan layer, which is then degraded by the enzyme^55^. Expression of the holin-lys cassette in the H111 *Δlys1,lys2,lys3* mutant strain caused cells lysis and concomitant release of eDNA and MVs (Fig. 3). These results show that the induction of endolysin-triggered cell lysis is required for eDNA release and is not affected by the killing activity of the antibiotic.

### Prophage induction enhances biofilm streamer formation in *B. cenocepacia* H111

**Fig. 4:**
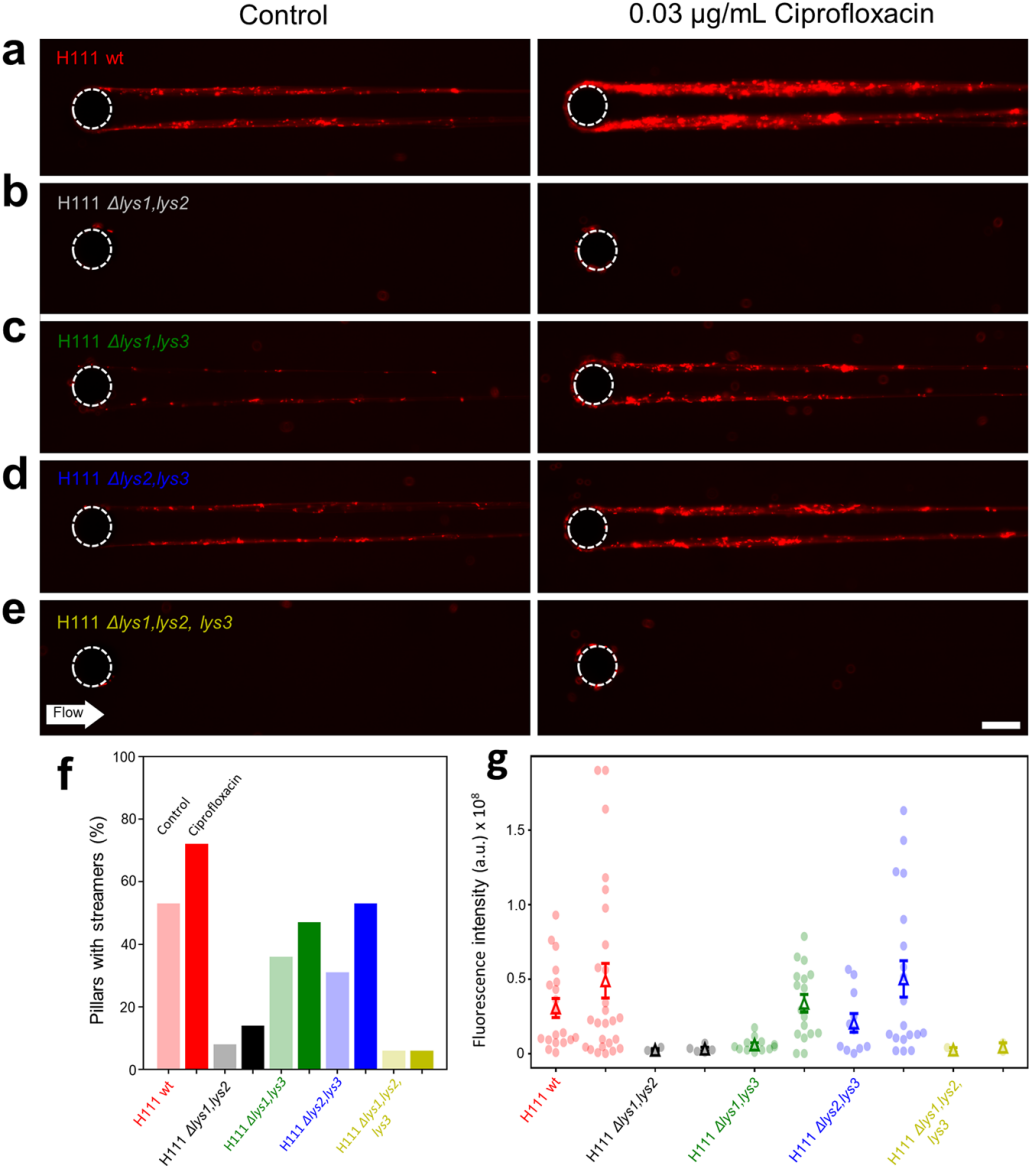
The role of endolysins in streamer formation. **a**–**e** Representative red fluorescence (eDNA) images of the biofilm streamers formed by *Burkholderia cenocepacia* H111 wt (**a**), H111 *Δlys1,lys2* (**b**), H111 *Δlys1,lys3* (**c**), H111 *Δlys2,lys3* (**d**) and H111 *Δlys1,lys2,lys3* (**e**) strains attached to a 50-µm pillar after 15 h of continuous flow of a diluted bacterial suspension at *U* = 2 mm/s, containing CPFLX at concentrations 0 ng mL^−1^ (left) and 300 ng mL^−^^1^ (right). The images were taken at channel mid-depth and are representative of the streamer morphology obtained with the different mutant strains. Scale bar 25 μm. **f** Percentage of 50-µm pillars (%) where streamers were formed after 15 h of a continuous flow of a diluted bacterial suspension at *U* = 2 mm/s, containing CPFLX at concentrations of 0 ng mL^−1^ (light coloured bars, left) and 300 ng mL^−1^ (coloured bars, right). The percentage is calculated over 36 pillars. **g** Red fluorescence intensity of the streamers on each pillar, *I*_*str*_ (points), and average red fluorescence intensity of the streamers, 〈*I*_*str*_〉 (triangles), measured after 15 h of a continuous flow of different *B. cenocepacia* H111 strains with CPFLX at concentrations of 0 ng mL^−1^ (left) and 300 ng mL^−1^ (right). The average red fluorescence intensity is calculated on the formed streamers, whose number is different depending on the strain (**f**). Error bars represent the standard error of the mean (SEM).

We used a microfluidic assay to test the influence of prophage induction on biofilm streamer formation in the H111 wildtype and the various endolysin mutants. In these experiments, we exclusively used CPFLX to induce explosive cell lysis and concomitant eDNA release, as CPFLX is of clinical relevance for the treatment of *B. cenocepacia* infections^56,57^ whereas MMC is not. As eDNA is an essential constituent of the streamer matrix, we visualized it by staining with PI (Fig. 4). The results of the streamer assay were consistent with the data obtained for eDNA and MV release: the wildtype, the H111 *Δlys1,lys3* and H111 *Δlys2,lys3* double mutants formed streamers, but H111 *Δlys1,lys2* or H111 *Δlys1,lys2,lys3* were not able to form them (Fig. 4a–e, left panel). An increase in eDNA accumulation on streamers was observed with the H111 wildtype, H111 *Δlys1,lys3* and H111 *Δlys2,lys3* when treated with CPFLX (Fig. 4a, c, d, right panel). The presence of CPFLX did not restore streamer formation of strains H111 *Δlys1,lys2* and H111 *Δlys1,lys2,lys3* (Fig. 4b, e, right panel). The difference in streamers formation between the different mutants could be quantified by the number of pillars where streamers were formed (Fig. 4f) and by the quantity of eDNA per unit length of the streamer, $\bar I_{{{{\mathrm{str}}}}}$, as assessed by the red fluorescence intensity of PI staining (Fig. 4g, Methods). The number of pillars colonized by the streamers, as well as the quantity of eDNA were significantly higher when the H111 wildtype or the double mutants H111 *Δlys1,lys3* and H111 *Δlys2,lys3* were treated with CPFLX, compared to the untreated controls (Fig. 4g). H111 not only formed streamers but also colonized the surface of the microfluid devices. However, no significant differences in bacterial surface coverage (Supplementary Fig. 3a) or eDNA release on the surface (Supplementary Fig. 3b) were observed between strains regardless of whether CPFLX was present or not.

### The SOS response in *B. cenocepacia* H111 is coupled with eDNA release and streamer formation

**Fig. 5:**
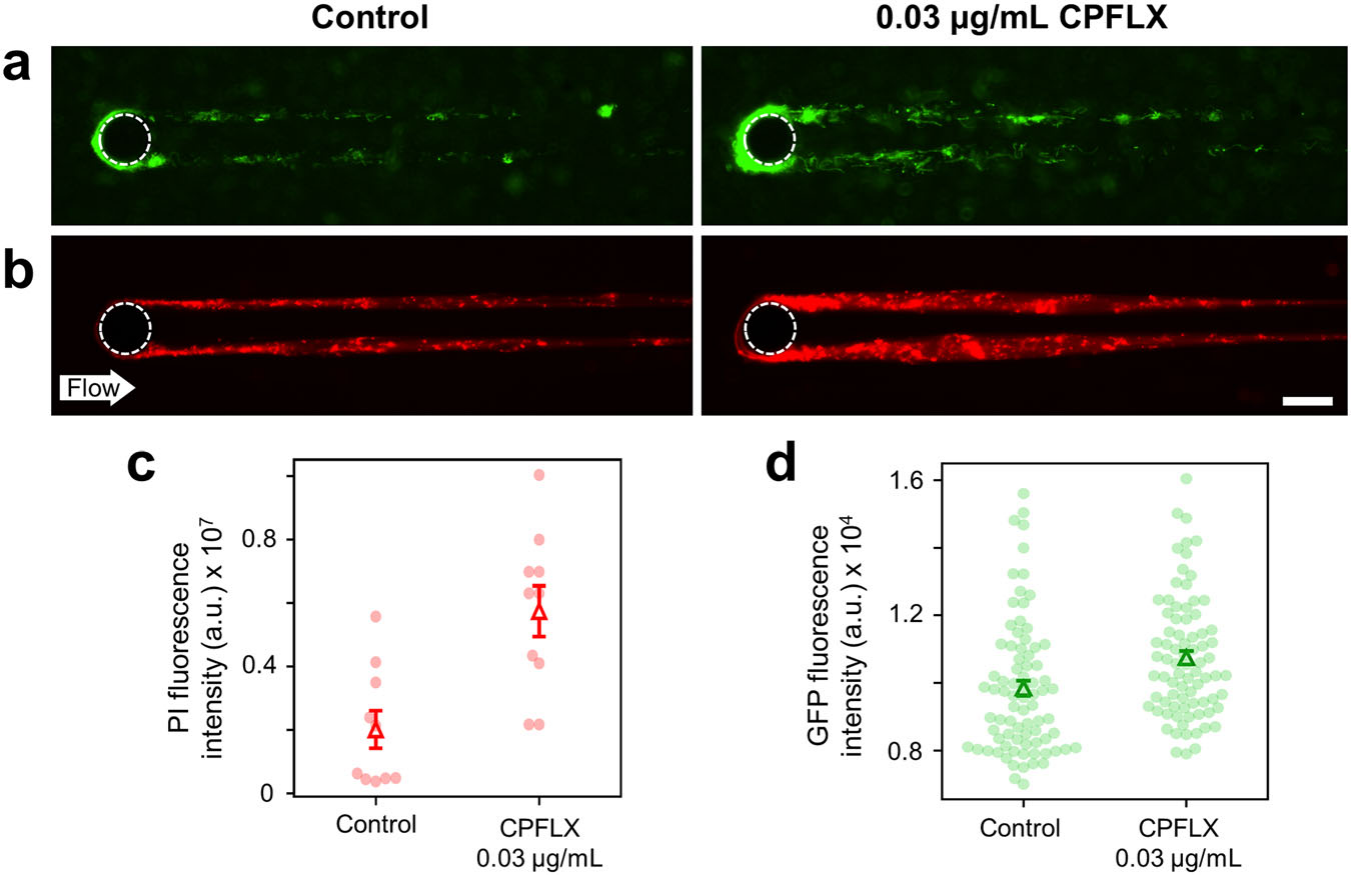
CPFLX induces the SOS response and stimulates streamer formation. **a**, **b** Representative GFP fluorescence (cells) and red fluorescence intensity (eDNA) images of the biofilm streamers formed by *B. cenocepacia* H111 pPROBE-*Pr_recA* cells attached to a 50-µm pillar after 15 h of a continuous flow of a diluted bacterial suspension at *U* = 2 mm/s, containing CPFLX at concentrations of 0 ng mL^−1^ (left) and 300 ng mL^−1^ (right). The images were taken at channel mid-depth and are representative of the streamer morphology. Scale bar 25 μm. **c** Red fluorescence intensity of the streamers, *I*_*str*_ (points), and average red fluorescence intensity of the streamers, 〈*I*_*str*_〉 (triangles), measured after 15 h of a continuous flow of different *B. cenocepacia* H111 pPROBE-*Pr_recA* cells with CPFLX at concentrations of 0 ng mL^−1^ (left) and 300 ng mL^−1^ (right). The average fluorescence intensity is calculated on 10 streamers. Error bars represent the standard error of the mean (SEM). **d** GFP fluorescence intensity of single cells (points) on the 10 different streamers of (**c**) and average GFP fluorescence intensity of a cell (triangles). Error bars represent the standard error of the mean (SEM).

The activation of the bacterial SOS response during streamer formation was investigated in the presence and absence of genotoxic stress by following the expression of a transcriptional fusion of *gfp* to the *recA* promoter. In liquid culture, the GFP signal increased approximately 3 and 1.1-fold in the presence of the DNA-damaging agents MMC and CPFLX, respectively (Supplementary Fig. 4). We next used this biosensor to monitor SOS induction in biofilms formed in our microfluidic device by measuring the fluorescence intensity over time. Even without the addition of a genotoxic agent, we observed a high level of *recA* expression in streamers (Fig. 5) as well as in the cells attached to the surface of microfluidic devices (Supplementary Fig. 5). This agrees with previous reports that showed that cells growing in biofilms experience stress, and as a consequence, a considerably higher proportion of the population is SOS induced when compared to planktonic cells^58,59^. Upon exposure to CPFLX, a 9% and 2% increased GFP signal was observed in streamers (Fig. 5) and cells attached to the microfluidic surface (Supplementary Fig. 5), respectively. This increase in GFP fluorescence intensity in the streamers was paralleled by an increase in the quantity of eDNA visualized by PI staining, indicating that stimulation of the SOS response in the presence of CPFLX is coupled to an increase of eDNA release, which in turn results in larger streamer structures.

## Discussion

**Fig. 6:**
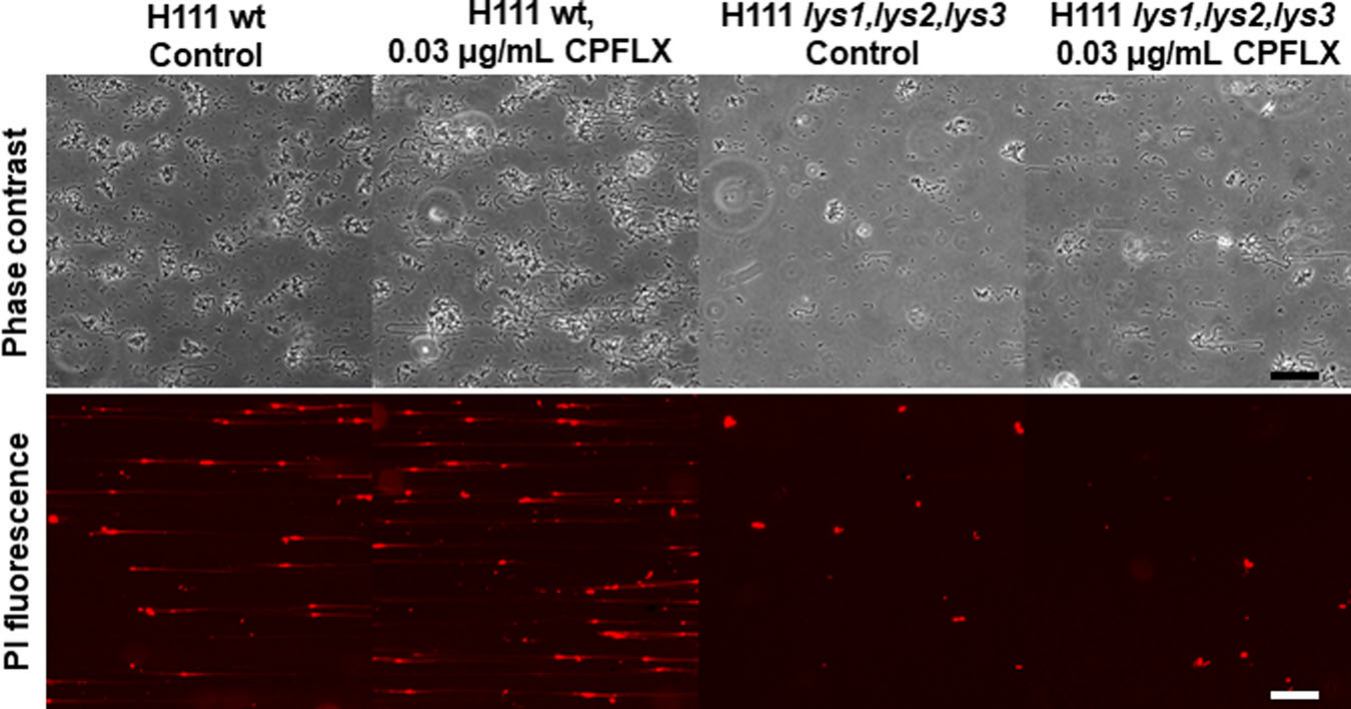
eDNA release of surface-grown microcolonies. **a**, **b** Representative phase contrast (upper panel) and red fluorescence (DNA, lower panel) images of the surface-attached *B. cenocepacia* H111 wt and H111 *Δlys1,lys2,lys3* cells attached to a glass surface after 15 h of a continuous flow of nutrients at *U* = 2 mm/s, containing CPFLX at concentrations of 0 ng mL^−^^1^ or 300 ng mL^−1^. Scale bar 50 μm.

Over the past few years, eDNA has emerged as an important biofilm matrix component. It is generally believed that, in most bacteria, the eDNA originates from cell lysis^19^. Given that induction of lysogenic prophages leads to DNA release from its host via explosive cell lysis^24,25^, and that DNA forms the backbone of biofilm streamers^14^, we speculated that prophages could play a crucial role in streamer formation. The key enzymes triggering explosive cell lysis are endolysins. These enzymes, which hydrolyse the peptidoglycan layer of the cell wall, are normally expressed at the late stage of the phage lytic cycle, as they are required for progeny escape^60^. To inactivate the lytic potential of the three prophages identified in the H111 genome, we not only deleted each of the three putative endolysins encoded by the three prophage regions but also generated various double mutants as well as a triple mutant. To assess the contribution of each of the endolysins in cell lysis, we followed growth in liquid culture by measuring optical densities and determining CFUs. In accordance with previous studies demonstrating that prophage induction leads to a decrease in optical density^61^, we observed that in the presence of 200 ng mL^−1^ MMC, the *B. cenocepacia* triple endolysin mutant showed less cell lysis (i.e. reduction of optical density) than the H111 wildtype while the reduction of CFUs was comparable between the strains (Fig. 1). The decreased lysis of the triple endolysin mutant in the presence of MMC was also seen in the reduced amounts of eDNA and MVs released by the strain relative to the wildtype (Fig. 2a, b). We also observed that many cells of the H111 wildtype strain growing on the surface of our microfluidic devices were embedded in strings of eDNA, while PI staining of the triple endolysin mutant identified dead cells but very little eDNA strings resulting from cell lysis (Fig. 6). Moreover, we show that heterologous expression of an holin-endolysin cassette of *P. aeruginosa* in the H111 *Δlys1,lys2,lys3* mutant strain caused explosive cells lysis and concomitant release of eDNA and MVs (Fig. 3). Collectively, these results show that induction of endolysin-triggered cell lysis is required for eDNA release independent of the killing effect of the inducing genotoxic agent.

Inactivation of the three endolysins encoded by the prophage regions present in H111 as well as the construction of various double endolysin mutants revealed that, under the conditions used, only *lys1* and *lys2*, but not *lys3*, are required to trigger explosive cell lysis and consequently DNA and MVs release (Fig. 2a, b). This suggests that the prophage linked to the *lys3* endolysin gene is silent or non-functional. This hypothesis is supported by the finding that the phage structures detected in the supernatant of the wildtype strain could be linked to the prophages connected to the endolysins *lys1* and *lys2* (Fig. 2c). Prophage region 3, which encodes Lys1, was previously reported to encode the myovirus ϕH111-1, which was reported to have a broad host range against many clinical isolates of *B. cenocepacia* and a relatively narrow one against other *Burkholderia* strains^51^. Although we did not detect a complete myrovirus in this study (Fig. 2c), one of the observed phage tails showed a similar structure as the one reported for ϕH111-1^51^.

While eDNA is widely recognized as an important component of the matrix of surface-grown biofilms in many species^17,20,62^, it was only recently that eDNA was found to be a critical structural element of *P. aeruginosa* biofilm streamers^14^. Here, we show that eDNA is also an essential component of *B. cenocepacia* streamers and that their formation depends on two prophages (present in regions 1 and 3; Supplementary Tables 1 and 2), which upon induction cause explosive cell lysis and concomitant eDNA release. While the importance of prophages in the development of surface-associated biofilms is well documented^24,25,63,64^, additional work will be required to confirm that prophages are more widely required for streamer formation.

Streamer formation caused by prophage induction in H111 is coupled with the bacterial SOS response, as a significantly higher *recA* promoter activity was detected under CPFLX treatment than in the untreated condition (Fig. 5). This observation is in line with previous studies, in which a link between prophage induction and the activation of the bacterial SOS response was reported^36,65^. The increase in *recA* promoter activity under CPFLX treatment in streamers and surface-attached cells is significant compared to the untreated control, but very low (Fig. 5 and Supplementary Fig. 5). The results obtained in growth curves suggest that these differences might be low because CPFLX is a weaker SOS-inducing agent than MMC (Supplementary Fig. 4). Besides, bacteria in flow experience a high level of stress that may result in a high background of *recA* promoter activation, which is supported by a recent study in which shear rate in microfluidic devices generates a sustained stress response in *P. aeruginosa*^66^.

In summary, this study demonstrates that prophage induction is essential for streamer formation in the opportunistic pathogen *B. cenocepacia* H111. Furthermore, we show that genotoxic compounds triggering the bacterial SOS response stimulate streamer formation via explosive cell lysis and concomitant eDNA release. Interestingly, the results obtained in this study suggest that prophages not only can boost the effects of antibiotics in bacterial populations by enhancing explosive cell lysis, but at the same time also enhance biofilm formation, in which cells are known to be more resistant to antibiotics^67^. However, although streamers are considered biofilm structures, it is currently unknown whether streamer cells are similarly resistant to stress as cells in surface-attached biofilms. In any case, our data suggest that care must be taken in the choice of antibiotics for treating bacterial infections, as SOS-inducing antibiotics used at concentrations that are insufficient to kill the entire bacterial population may not only promote biofilm formation but also the formation of MVs that protect the cells from certain antibiotics^68–70^.

## Methods

### Bacterial strains, plasmids and growth conditions

The bacterial strains, plasmids, and primers used in this study are listed in Supplementary Tables 3–5, respectively. *Escherichia coli* was used as a host for the plasmid constructs and was routinely grown on Lysogeny Broth (LB, Difco, 240210) at 37 °C and 220 rpm. *B. cenocepacia* H111 cultures were grown in LB at 37 °C and 220 rpm. *Pseudomonas* sp. IsoF (IsoF) was used as a control in some experiments and was grown at 30 °C and 220 rpm. For mutant selection or transconjugants, *Pseudomonas* isolation agar (PIA, Difco, 292710) was used. Growth curves were done in 100 ml flasks with 30 ml of LB. The flasks were initially inoculated at 0.01 a.u (OD600) and grown for 4 h before treatment with MMC or CPFLX. Treatment with mitomycin C (MMC) or ciprofloxacin (CPFLX) lasted 4 h (total growth time: 8 h). To obtain a rough estimate of the sensitivity of H111 to MMC and CPFLX, the minimum inhibitory concentrations (MICs) were determined by classic broth dilution assays in LB medium. Growth in the presence of different concentrations of MMC and CPFLX was measured as OD600 nm after 24 h of growth at 37 °C in a 96-well plate. The determined MIC concentrations were 200 ng mL^−1^ for MMC and 150 ng mL^−1^ for CPFLX.

The pJN105*hol_lys* plasmid for expression of the holin PA0614 and the endolysin PA0629 (Lys) of *P. aeruginosa* PAO1 (PAO1) were constructed as follows: The two genes were PCR amplified using primers PA0614_F and PA0629H_R and cloned into the BamHI site downstream of the arabinose-inducible promoter on plasmid pJN105. The resulting plasmid was introduced into H111 *Δlys1,lys2,lys3* by triparental conjugation using pRK2013 as helper plasmid. Induction was accomplished by adding a 2% arabinose solution to an exponentially growing culture, following incubation for additional 20 h. If applicable and not otherwise specified, antibiotics were added to the following final concentrations: 100 μg mL^−1^ trimetrophim (Tp), 20 μg mL^−1^ gentamycin (Gm), 50 μg mL^−1^ kanamycin (Km), 200 ng mL^−1^ mitomycin C (MMC) and 300 ng mL^−1^ ciprofloxacin (CPFLX).

### Bioinformatics

Prophages were re-identified and re-localized in the genome of *B. cenocepacia* H111 using PHASTER (PHAge Search Tool – Enhanced Release). This web server allows the identification and annotation of prophage sequences within bacterial genomes. PHASTER scores prophage regions as intact (score > 90), questionable (score 70–90), or incomplete (score < 70). For more information regarding score calculation, see^52^^,^^53^.

### Strain manipulation

Genes encoding for putative hydrolases and/or peptidoglycan binding proteins were selected for mutagenesis to create endolysin mutants. For mutagenesis, 500–1000 bp flanking the gene selected for deletion were cloned into the suicide plasmid pGPI-SceI, which carries a Tp resistance cassette. This plasmid was introduced in *B. cenocepacia* H111 via triparental conjugation and integrated into the genome by a single recombination event. Then, in a second conjugation step, the pDAI-SceI plasmid was introduced into the strain, after which the I-SceI endonuclease creates a double strand break in the chromosome that stimulates the second recombination event. This second recombination event results in either the wild type or the mutant genotype. The pRK2013 helper plasmid was used to provide the genes encoding the conjugation machinery. In frame deletion mutants were verified by PCR and sequencing, and the pDAI-SceI plasmid was cured. All primers and restriction enzymes used for cloning and to check deletions are listed in Supplementary Table 5.

### Extracellular particle quantification

Previous work has shown that endolysin-triggered explosive cell lysis not only leads to the release of eDNA but also to the formation of membrane vesicles (MVs) as a consequence of the self-annealing of membrane fragments^26,71^. Hence, both eDNA release and MV formation are indicative of explosive cell lysis and both parameters were used in our study to evaluate the specific contributions of each of the H111 prophages. For MV quantification, cell cultures were grown in 250 ml flasks with 80 ml of LB for 8 h at 37 °C and 220 rpm. The cultures were inoculated with an initial cell density of 0.01 a.u at OD600_nm_. If applicable, mitomycin C (MMC) was added 4 h after inoculation to a final concentration of 200 ng mL^−1^. After 8 h of growth, cell cultures were centrifuged at 8000 rpm at 4 °C for 30 min and supernatants were filtered through a 0.45 µm Durapore Membrane Filter (hydrophilic PVDF; Merck). The extracellular particles were quantified directly from filtrates using Nanosight NS300 (Malvern Panalytical), a device used for nanoparticle tracking analysis (NTA). For NTA, samples were diluted to a final concentration of 20–100 particles/frame using HyClone HyPure Water (GE). Maximum camera detection sensitivity was set, and the processing threshold was set at 5. Three independent experiments were performed, and each experiment represents the mean value of 15 technical replicates.

### TEM samples

To prepare samples for phage particle observation under TEM, cell-free filtrates obtained in section “Extracellular particle quantification” were used for ultracentrifugation at 150,000*g* at 4 °C for 90 min, and pellets were resuspended in double distilled water (ddH_2_O). Before sample observation, formvar-coated 300-mesh copper grids (Electron Microscopy Sciences, USA) were glow discharged, and samples were diluted 10 times in ddH_2_O. Then, the diluted samples were stained with uranyl acetate (UA). For this purpose, the shiny side of the grid was inoculated with 5 μl of the diluted sample for 1 min. Carefully, the grid is put over a UA drop (1%) on parafilm and incubated for 1 min. Then, the grids were dried with filter paper. The samples were visualized using FEI Tecnai G2 Spirit TEM (FEI, Hillsboro, USA) at 120 kV acceleration voltage with the detector side-mounted digital camera Gatan Orius 1000 (4k × 2.6k pixels). Protein quantification (Pierce^TM^ BCA Protein Assay Kit, Thermo Fisher) and lipid quantification (FM™1–43, Invitrogen) of the samples obtained after ultracentrifugation indicate that the extracellular particles that were quantified mostly correspond to membrane vesicles (MVs) (Supplementary Fig. 6).

### DNA quantification in supernatants

DNA was quantified in cell-free filtrates obtained in section “Extracellular particle quantification” using the SYTOX fluorescent dye (Life Technologies, USA). For sample preparation, cell-free supernatants were mixed with a 1000× dilution of SYTOX at a 1:10 ratio, respectively. Then, SYTOX fluorescence was measured with a Synergy HT microplate reader (MWGt Sirius HT, Biotek, Germany) using λ485 nm for excitation and λ528 nm for emission.

### Live cell imaging of DNA release

To observe cell lysis and DNA release, agarose pads were prepared for microscopy. For this, 8–9 mm Ø × 1 mm depth adhesive silicon isolators (Grace BioLabs, JTR8R-1.0) were attached to microscope slides and filled with 64 μL ABC medium with 0.7% agar supplemented with propidium iodide (PI, 250 ng/ml) and MMC (200 ng/ml). Bacterial overnight cultures were washed with NaCl 0.9% and adjusted to a final concentration of 1 a.u. The agar pads were inoculated with 1 μL of the adjusted culture and a cover slip was placed on top after the inoculant had dried. Agarose pads were incubated at room temperature overnight, and the next day cell lysis was monitored every 20 min for a few hours. The focus had to be frequently re-adjusted due to evaporation of the medium. Image acquisition was done using an epifluorescence microscope DM6000B (Leica). Image analysis was performed with Fiji-Image^72^.

### Microfluidics

Cell suspensions were prepared by inoculating 3 mL ABC medium with cells from a frozen stock and incubating overnight at 37 °C, while shaking at 200 rpm. 30 µL of overnight suspension were then inoculated in 3 mL ABC medium and incubated for 6 h at 37 °C, while shaking at 200 rpm. The suspensions were then diluted in fresh Tryptone Broth to final optical density OD_600_ < 0.01. Propidium iodide, PI (Sigma Aldrich) was added to the medium to a final concentration of 2 μg mL^−1^ for eDNA visualization. For antibiotic treatment experiments, ciprofloxacin (Sigma Aldrich) was first dissolved in 0.1 N HCl to a final concentration of 20 mg mL^−1^ and then further diluted in Tryptone broth (TB) to obtain solutions of final concentration 300 ng mL^−1^. To analyse streamer formation in flow and visualize surface-attached *B. cenocepacia* cells, a polydimethylsiloxane (PDMS) microfluidic device was used. The device had eight (or twelve) channels on the same chip, each containing six cylindrical pillars of diameter 50 μm. The channel was 40 μm high and 1 mm wide. Pillars were located at the centre of the channel and the distance between pillars was 5 mm. The flow was driven by a syringe pump (neMESYS 290 N, CETONI, Germany) with a constant flow rate of *Q* = 0.3 mL/h, corresponding to a mean flow velocity of 2.1 mm/s as in reference^14^. Additional information on the hydrodynamic parameters of the microfluidic platform can be found in reference^73^. Prior to each experiment, all microfluidic channels were washed with 2 mL of TB medium. To analyse streamers formation in flow, a diluted bacteria suspension (OD_600_ < 0.01; cell concentration < 10^6^ cells/mL) was flown for 20 h, at room temperature (*T* = 20 ± 0.5 °C). To visualize surface-attached *B. cenocepacia* cells, the microfluidic channel was inoculated with a diluted bacterial suspension (OD600 = 0.05; cell concentration < 5 × 10^6^ cells/mL), cells were incubated in the channel for 1 h without flow to promote the surface-attachment and, subsequently, fresh TB medium was flown with a constant flow rate of *Q* = 0.3 mL/h for 20 h. All imaging was performed on an inverted microscope (Ti-Eclipse, Nikon, Japan) using a digital camera (ORCA-Flash4.0 V3 Digital CMOS camera, Hamamatsu Photonics, Japan). Phase-contrast microscopy (20× magnification) was used to image bacterial cells, while epifluorescence microscopy was used to quantify eDNA concentration.

### Statistical analysis

All image analyses were performed in Fiji-Image^72^. All the experiments were repeated three times with consistent results and two technical replicates were present in each experiment. The average fluorescence intensity of the streamers in each channel was calculated on the red fluorescence signal as $I_{{{{\mathrm{str}}}}} = \Sigma \left( {I_{{{{\mathrm{px}}}}} - I_{{{{\mathrm{th}}}}}} \right),$ where *I*_px_ is the intensity of the pixels, and *I*_th_ is a threshold calculated as 1.2 times the average intensity of the background, measured in the region of the channel on the side of the streamer, as in reference^14^. The sum was performed on a 550 µm × 100 µm area located downstream of each pillar. The average red fluorescence intensity of the streamers 〈*I*_*str*_ 〉 is obtained as the average of *I*_*str*_. over all the pillars where streamers are formed. During each experiment, two channels with the same conditions were present.

### Supplementary information

Supplementary Material

nr-reporting-summary

Video 1

SupplementaryVideo

## Data Availability

The data that support the findings of this study are available from the corresponding author upon reasonable request.
