# Supplementary material for: Genotoxic stress stimulates eDNA release via explosive cell lysis and thereby promotes streamer formation of Burkholderia cenocepacia H111 cultured in a microfluidic device
Source: NPJ Biofilms Microbiomes. 2023 Dec 9;9:96. doi: 10.1038/s41522-023-00464-7 (PMC10710452; doi:10.1038/s41522-023-00464-7)
Supplement: Supplementary file 1 — Supplementary Material [file 41522_2023_464_MOESM1_ESM.docx]

- **Supplementary Material:**

**Supplementary Table 1.** Prophages in *Burkholderia cenocepacia* H111. PHASTER score is indicated in brackets.

| **Region** | **Endolysin** | **Length (kb)** | **Completeness** | **Position** | **N° proteins** | **Phage-hit proteins** | **GC (%)** | **Comments** |
| --- | --- | --- | --- | --- | --- | --- | --- | --- |
| 1 | Lys2 | 22.9 | Intact (100) | 100107-123014 | 28 | 27 | 68.79 | - |
| 2 | Lys3 | 64.5 | Intact (150) | 1582204-1646740 | 80 | 47 | 62.65 | - |
| 3 | Lys1 | 36 | Intact (140) | 2592369-2628371 | 43 | 33 | 63.28 | ϕH111-1 (myovirus) |

**Supplementary Table 2.** Prophage regions of *Burkholderia cenocepacia* H111.

| **Region 1** | **CDS Position** | **BLAST Hit** | **E-Value** |
| --- | --- | --- | --- |
| 1 | 100107..100137 | attL | 0 |
| 2 | complement(100206..101282) | PHAGE_Burkho_phiE202_NC_009234: gp10, site-specific recombinase, phage integrase family; I35_RS00425; phage(gi134288747) | 1.68E-83 |
| 3 | complement(101609..101980) | hypothetical protein; I35_RS34660 | 0 |
| 4 | complement(101977..104769) | PHAGE_Burkho_phiE202_NC_009234: gp11, conserved hypothetical protein; I35_RS00430; phage(gi134288779) | 0 |
| 5 | complement(104775..105032) | PHAGE_Burkho_phiE202_NC_009234: gp12, hypothetical protein; I35_RS00435; phage(gi134288787) | 6.94E-16 |
| 6 | complement(105160..105408) | PHAGE_Burkho_phiE202_NC_009234: gp19, conserved hypothetical protein; I35_RS00440; phage(gi134288771) | 1.57E-49 |
| 7 | complement(105590..105805) | PHAGE_Burkho_phiE202_NC_009234: gp20, conserved hypothetical protein; I35_RS00445; phage(gi134288764) | 5.11E-10 |
| 8 | 105968..106387 | PHAGE_Burkho_phiE202_NC_009234: gp21; I35_RS00450; phage(gi134288765) | 2.44E-08 |
| 9 | 106705..107001 | PHAGE_Burkho_KL3_NC_015266: gp14; I35_RS00455; phage(gi327198059) | 2.00E-18 |
| 10 | complement(107189..108259) | PHAGE_Burkho_phiE202_NC_009234: gp24, fels-2 prophage protein; I35_RS00460; phage(gi134288767) | 1.15E-168 |
| 11 | complement(108256..108687) | PHAGE_Burkho_phiE202_NC_009234: gp25, fels-2 prophage protein; I35_RS00465; phage(gi134288777) | 1.73E-58 |
| 12 | complement(108710..111280) | PHAGE_Burkho_phiE202_NC_009234: gp26, bacteriophage membrane protein; I35_RS00470; phage(gi134288775) | 1.20E-171 |
| 13 | complement(111296..111409) | PHAGE_Burkho_phiE202_NC_009234: gp27, phage tail protein, P2 GpE family; I35_RS34665; phage(gi134288776) | 1.11E-14 |
| 14 | complement(111418..111789) | PHAGE_Burkho_phiE202_NC_009234: gp28, phage tail protein E; I35_RS00480; phage(gi134288780) | 3.68E-39 |
| 15 | complement(111865..112368) | PHAGE_Burkho_phiE202_NC_009234: gp29, phage major tail tube protein; I35_RS00485; phage(gi134288759) | 6.42E-84 |
| 16 | complement(112403..113575) | PHAGE_Burkho_phiE202_NC_009234: gp30, phage tail sheath protein; I35_RS00490; phage(gi134288772) | 0 |
| 17 | complement(113630..114328) | PHAGE_Burkho_phiE202_NC_009234: gp31, bacteriophage-acquired protein; I35_RS00495; phage(gi134288758) | 1.58E-104 |
| 18 | complement(114345..116915) | PHAGE_Burkho_phiE202_NC_009234: gp32, bacteriophage protein; I35_RS00500; phage(gi134288773) | 5.42E-161 |
| 19 | complement(116922..117464) | PHAGE_Burkho_phiE202_NC_009234: gp33, phage tail protein I; I35_RS00505; phage(gi134288744) | 8.01E-81 |
| 20 | complement(117457..118371) | PHAGE_Burkho_phiE202_NC_009234: gp34, phage baseplate assembly protein; I35_RS00510; phage(gi134288785) | 8.39E-132 |
| 21 | complement(118368..118730) | PHAGE_Burkho_phiE202_NC_009234: gp35, phage baseplate assembly protein; I35_RS00515; phage(gi134288768) | 3.24E-54 |
| 22 | complement(118727..119413) | PHAGE_Burkho_phiE202_NC_009234: gp36, gpV; I35_RS00520; phage(gi134288750) | 1.01E-106 |
| 23 | complement(119923..120372) | PHAGE_Burkho_phiE202_NC_009234: gp40, P2 phage tail completion protein R (GpR); I35_RS00525; phage(gi134288754) | 4.26E-36 |
| 24 | complement(120365..120535) | PHAGE_Burkho_phiE202_NC_009234: gp41, LysC; I35_RS34670; phage(gi134288745) | 4.75E-13 |
| 25 | complement(120489..120929) | PHAGE_Burkho_phiE202_NC_009234: gp42, protein LysB; I35_RS00530; phage(gi134288770) | 1.01E-18 |
| 26 | complement(120926..121783) | PHAGE_Burkho_phiE202_NC_009234: gp43, bacteriophage-acquired protein; I35_RS00535; phage(gi134288769) | 2.96E-114 |
| 27 | complement(121780..122046) | PHAGE_Burkho_phiE202_NC_009234: gp44, putative bacteriophage membrane protein; I35_RS00540; phage(gi134288752) | 8.75E-20 |
| 28 | complement(122048..122392) | PHAGE_Burkho_phiE202_NC_009234: gp45, putative bacteriophage membrane protein; I35_RS00545; phage(gi134288749) | 1.62E-49 |
| 29 | complement(122409..122615) | PHAGE_Burkho_phiE202_NC_009234: gp46, phage Tail Protein X; I35_RS00550; phage(gi134288782) | 1.50E-23 |
| 30 | 123014..123044 | attR | 0 |
| **Region 2** | **CDS Position** | **BLAST Hit** | **E-Value** |
| 1 | 1582204..1582216 | attL | 0 |
| 2 | 1589281..1589294 | attL | 0 |
| 3 | 1593686..1594498 | PHAGE_Bacill_G_NC_023719: gp243; I35_RS07320; phage(gi593777699) | 1.08E-22 |
| 4 | 1594495..1595457 | PHAGE_Bacill_G_NC_023719: gp245; I35_RS07325; phage(gi593777701) | 7.17E-43 |
| 5 | 1595515..1595730 | transporter; I35_RS07330 | 0 |
| 6 | 1595875..1596072 | hypothetical protein; I35_RS07335 | 0 |
| 7 | 1596128..1596373 | hypothetical protein; I35_RS07340 | 0 |
| 8 | complement(1596597..1596863) | PHAGE_Burkho_AH2_NC_018283: excisionase; I35_RS07345; phage(gi399529070) | 4.60E-36 |
| 9 | complement(1596863..1597543) | PHAGE_Pseudo_F116_NC_006552: hypothetical protein; I35_RS07350; phage(gi56692934) | 1.17E-38 |
| 10 | complement(1597540..1597779) | hypothetical protein; I35_RS07355 | 0 |
| 11 | complement(1597776..1598291) | hypothetical protein; I35_RS07360 | 0 |
| 12 | complement(1598288..1598671) | PHAGE_Burkho_Bcep22_NC_005262: hypothetical protein; I35_RS07365; phage(gi38640354) | 8.32E-45 |
| 13 | complement(1598668..1598886) | PHAGE_Caulob_Cr30_NC_025422: hypothetical protein; I35_RS07370; phage(gi725949259) | 1.84E-17 |
| 14 | complement(1598931..1599311) | PHAGE_Gordon_Yvonnetastic_NC_031230: tail tube protein; I35_RS07375; phage(gi100121) | 2.46E-31 |
| 15 | complement(1599311..1599895) | PHAGE_Pseudo_vB_PaeP_Tr60_Ab31_NC_023575: Hypothetical protein; I35_RS34500; phage(gi589286899) | 3.77E-13 |
| 16 | complement(1599898..1600434) | hypothetical protein; I35_RS07385 | 0 |
| 17 | complement(1600427..1600744) | hypothetical protein; I35_RS35040 | 0 |
| 18 | complement(1600741..1601709) | PHAGE_Achrom_JWF_NC_029075: hypothetical protein; I35_RS34505; phage(gi985758700) | 3.52E-21 |
| 19 | complement(1601717..1602052) | PHAGE_Pseudo_phiPSA1_NC_024365: hypothetical protein; I35_RS07395; phage(gi658307727) | 3.23E-07 |
| 20 | complement(1602071..1602355) | hypothetical protein; I35_RS07400 | 0 |
| 21 | complement(1602352..1603077) | PHAGE_Strept_phiARI0460_1_NC_031913: hypothetical protein; I35_RS35045; phage(gi100040) | 1.65E-15 |
| 22 | complement(1603090..1604085) | PHAGE_Bacill_BalMu_1_NC_030945: hypothetical protein; I35_RS07405; phage(gi100053) | 4.07E-48 |
| 23 | complement(1604417..1604794) | hypothetical protein; I35_RS07410 | 0 |
| 24 | complement(1604809..1605276) | hypothetical protein; I35_RS07415 | 0 |
| 25 | complement(1605307..1605669) | hypothetical protein; I35_RS07420 | 0 |
| 26 | complement(1605860..1606075) | PHAGE_Burkho_Bcep176_NC_007497: gp41; I35_RS07425; phage(gi77864666) | 4.65E-21 |
| 27 | complement(1606075..1606440) | hypothetical protein; I35_RS07430 | 0 |
| 28 | complement(1606416..1606640) | hypothetical protein; I35_RS07435 | 0 |
| 29 | complement(1606814..1607179) | hypothetical protein; I35_RS07440 | 0 |
| 30 | complement(1607231..1607449) | PHAGE_Burkho_phi1026b_NC_005284: gp52; I35_RS07445; phage(gi38707942) | 1.00E-16 |
| 31 | complement(1607647..1607844) | hypothetical protein; I35_RS07450 | 0 |
| 32 | complement(1608523..1608936) | hypothetical protein; I35_RS07455 | 0 |
| 33 | complement(1608944..1609636) | PHAGE_Pseudo_PS_1_NC_029066: CI repressor; I35_RS35050; phage(gi985757672) | 2.19E-13 |
| 34 | 1609707..1609973 | hypothetical protein; I35_RS35055 | 0 |
| 35 | complement(1610138..1610416) | hypothetical protein; I35_RS07465 | 0 |
| 36 | 1610563..1610862 | PHAGE_Burkho_KS5_NC_015265: gp9; I35_RS07470; phage(gi327198007) | 1.22E-21 |
| 37 | 1610961..1611260 | PHAGE_Escher_ECBP5_NC_027330: hypothetical protein; I35_RS07475; phage(gi849119779) | 4.27E-05 |
| 38 | 1611304..1611621 | PHAGE_Burkho_BcepF1_NC_009015: hypothetical protein; I35_RS07480; phage(gi126010941) | 2.53E-41 |
| 39 | 1611631..1612515 | PHAGE_Burkho_phiE125_NC_003309: putative chromosome partitioning protein; I35_RS07485; phage(gi17975220) | 4.18E-123 |
| 40 | 1612512..1613366 | PHAGE_Arthro_KellEzio_NC_031231: hypothetical protein; I35_RS35060; phage(gi100040) | 2.79E-06 |
| 41 | 1613371..1614051 | hypothetical protein; I35_RS34520 | 0 |
| 42 | 1614048..1614593 | PHAGE_Burkho_phi6442_NC_009235: gp61; I35_RS07500; phage(gi134288657) | 6.10E-41 |
| 43 | 1614606..1614947 | MarR family transcriptional regulator; I35_RS07505 | 0 |
| 44 | 1614944..1615387 | PHAGE_Burkho_Bcep176_NC_007497: gp5; I35_RS07510; phage(gi77864630) | 3.35E-93 |
| 45 | 1615390..1615722 | PHAGE_Burkho_phi1026b_NC_005284: gp75; I35_RS07515; phage(gi38707965) | 3.77E-48 |
| 46 | 1615719..1615958 | PHAGE_Burkho_phi6442_NC_009235: gp65; I35_RS07520; phage(gi134288623) | 7.77E-20 |
| 47 | 1615958..1616215 | PHAGE_Burkho_Bcep22_NC_005262: hypothetical protein; I35_RS07525; phage(gi38640326) | 3.01E-17 |
| 48 | 1616269..1616700 | hypothetical protein; I35_RS35065 | 0 |
| 49 | 1617125..1617817 | PHAGE_Rhodob_RcRhea_NC_028954: minor tail protein; I35_RS07530; phage(gi971760207) | 1.93E-22 |
| 50 | 1617979..1618557 | hypothetical protein; I35_RS07535 | 0 |
| 51 | 1618676..1620571 | PHAGE_Vibrio_VP58.5_NC_027981: gp02 protein; I35_RS07540; phage(gi937456073) | 2.12E-123 |
| 52 | 1620613..1620849 | phage head-tail adapter protein; I35_RS07545 | 0 |
| 53 | 1620807..1622501 | PHAGE_Burkho_AH2_NC_018283: portal protein; I35_RS07550; phage(gi399529104) | 3.88E-77 |
| 54 | 1622523..1623419 | PHAGE_Burkho_phiE125_NC_003309: putative capsid assembly protein/protease; I35_RS07555; phage(gi17975166) | 2.44E-53 |
| 55 | 1623441..1624028 | hypothetical protein; I35_RS07560 | 0 |
| 56 | 1624056..1624469 | PHAGE_Bacter_Lily_NC_028841: hypothetical protein; I35_RS07565; phage(gi971748258) | 1.68E-17 |
| 57 | 1624542..1625588 | PHAGE_Burkho_BcepNazgul_NC_005091: capsid protein E; I35_RS07570; phage(gi34610166) | 8.51E-50 |
| 58 | 1625597..1625833 | hypothetical protein; I35_RS07575 | 0 |
| 59 | 1625837..1626181 | hypothetical protein; I35_RS07580 | 0 |
| 60 | 1626174..1626776 | hypothetical protein; I35_RS34525 | 0 |
| 61 | 1626789..1626968 | DUF2635 domain-containing protein; I35_RS07590 | 0 |
| 62 | 1626965..1628455 | PHAGE_Entero_SfI_NC_027339: tail sheath protein; I35_RS07595; phage(gi849250296) | 1.42E-137 |
| 63 | 1628525..1628899 | PHAGE_Salmon_118970_sal3_NC_031940: hypothetical protein; I35_RS07600; phage(gi100017) | 7.46E-06 |
| 64 | 1628903..1629463 | hypothetical protein; I35_RS07605 | 0 |
| 65 | 1629627..1631051 | PHAGE_Shigel_SfIV_NC_022749: tail/DNA circulation protein; I35_RS07610; phage(gi557307540) | 7.07E-34 |
| 66 | 1631068..1632717 | PHAGE_Burkho_BcepB1A_NC_005886: gp14 T; I35_RS07615; phage(gi72257065) | 2.42E-09 |
| 67 | 1632717..1633859 | PHAGE_Shigel_SfII_NC_021857: tail protein; I35_RS07620; phage(gi526244652) | 7.60E-32 |
| 68 | 1633904..1634422 | PHAGE_Salmon_118970_sal3_NC_031940: siderophore-interacting protein; I35_RS07625; phage(gi100025) | 3.73E-35 |
| 69 | 1634426..1634872 | PHAGE_Escher_D108_NC_013594: hypothetical protein; I35_RS07630; phage(gi281199691) | 1.05E-31 |
| 70 | 1634874..1636037 | PHAGE_Salmon_118970_sal3_NC_031940: hypothetical protein; I35_RS07635; phage(gi100027) | 1.72E-38 |
| 71 | 1636044..1636640 | PHAGE_Shigel_SfIV_NC_022749: tail protein; I35_RS07640; phage(gi557307545) | 2.82E-22 |
| 72 | 1637489..1638190 | PHAGE_Pseudo_PPpW_3_NC_023006: putative tail-collar fibre protein; I35_RS07645; phage(gi564292496) | 6.22E-35 |
| 73 | 1638187..1638381 | hypothetical protein; I35_RS07650 | 0 |
| 74 | 1638510..1639283 | hypothetical protein; I35_RS07655 | 0 |
| 75 | 1639434..1639616 | hypothetical protein; I35_RS07660 | 0 |
| 76 | 1639609..1640106 | PHAGE_Burkho_Bcep176_NC_007497: gp58; I35_RS07665; phage(gi77864683) | 8.99E-106 |
| 77 | 1640103..1640648 | PHAGE_Bacill_AR9_NC_031039: portal protein; I35_RS07670; phage(gi100032) | 2.19E-16 |
| 78 | 1640645..1641124 | PHAGE_Salmon_IME207_NC_031924: hypothetical protein; I35_RS07675; phage(gi100020) | 5.55E-14 |
| 79 | complement(1641236..1641613) | PHAGE_Pseudo_phi3_NC_030940: serine recombinase; I35_RS35070; phage(gi100001) | 6.41E-23 |
| 80 | complement(1642257..1643336) | PHAGE_Burkho_AH2_NC_018283: integrase; I35_RS07680; phage(gi399529076) | 2.11E-138 |
| 81 | 1643457..1644497 | tRNA dihydrouridine(20/20a) synthase DusA; I35_RS07685 | 0 |
| 82 | 1644552..1644627 | tRNA | 0 |
| 83 | 1644659..1644672 | attR | 0 |
| 84 | 1644847..1645095 | hypothetical protein; I35_RS07695 | 0 |
| 85 | 1645618..1646133 | PHAGE_Erwini_vB_EamM_Phobos_NC_031043: integrase/recombinase; I35_RS07700; phage(gi100097) | 3.15E-05 |
| 86 | 1646740..1646752 | attR | 0 |
| **Region 3** | **CDS Position** | **BLAST Hit** | **E-Value** |
| 1 | complement(2592369..2594318) | PHAGE_Bacill_G_NC_023719: gp245; I35_RS11935; phage(gi593777701) | 8.69E-14 |
| 2 | complement(2594585..2594989) | rod shape-determining protein RodA; I35_RS11940 | 0 |
| 3 | complement(2595001..2595189) | hypothetical protein; I35_RS35305 | 0 |
| 4 | 2595188..2595370 | rubredoxin; I35_RS11945 | 0 |
| 5 | complement(2596457..2597146) | PHAGE_Burkho_KS9_NC_013055: hypothetical protein gp28; I35_RS11950; phage(gi255033760) | 1.81E-77 |
| 6 | complement(2597186..2597527) | hypothetical protein; I35_RS11955 | 0 |
| 7 | 2597658..2598284 | PHAGE_Burkho_phiE125_NC_003309: hypothetical protein; I35_RS11960; phage(gi17975193) | 2.04E-57 |
| 8 | complement(2598315..2598899) | PHAGE_Ralsto_RSA1_NC_009382: hypothetical protein; I35_RS34595; phage(gi145708078) | 3.09E-28 |
| 9 | complement(2599044..2599655) | PHAGE_Ralsto_RSA1_NC_009382: hypothetical protein; I35_RS11970; phage(gi145708077) | 1.35E-11 |
| 10 | complement(2599543..2600013) | hypothetical protein; I35_RS35310 | 0 |
| 11 | complement(2600003..2600500) | PHAGE_Ralsto_RSA1_NC_009382: hypothetical protein; I35_RS11980; phage(gi145708076) | 4.48E-21 |
| 12 | complement(2600507..2601106) | PHAGE_Burkho_KL3_NC_015266: gp51; I35_RS35315; phage(gi327198096) | 4.55E-11 |
| 13 | complement(2601110..2601895) | PHAGE_Burkho_phiE125_NC_003309: DNA adenine methylase; I35_RS11985; phage(gi17975188) | 2.93E-166 |
| 14 | complement(2602173..2602820) | PHAGE_Rhodof_P26218_NC_029061: hypothetical protein; I35_RS11990; phage(gi985757613) | 8.18E-18 |
| 15 | complement(2602817..2603383) | PHAGE_Rhodof_P26218_NC_029061: hypothetical protein; I35_RS11995; phage(gi985757614) | 1.16E-65 |
| 16 | complement(2603380..2603832) | PHAGE_Pseudo_vB_PaeS_PAO1_Ab18_NC_026594: hypothetical protein; I35_RS12000; phage(gi764160572) | 4.22E-24 |
| 17 | complement(2603906..2604955) | PHAGE_Salmon_SP_004_NC_021774: tail protein; I35_RS12005; phage(gi526003638) | 2.80E-51 |
| 18 | complement(2604965..2605171) | PHAGE_Entero_P88_NC_026014: tail component protein; I35_RS12010; phage(gi744692729) | 1.78E-14 |
| 19 | complement(2605146..2606024) | PHAGE_Entero_Arya_NC_031048: XRE family transcriptional regulator; I35_RS12015; phage(gi100024) | 2.65E-22 |
| 20 | complement(2606035..2608476) | PHAGE_Haemop_SuMu_NC_019455: bacteriophage tail length determination protein; I35_RS12020; phage(gi418489066) | 5.67E-15 |
| 21 | complement(2608557..2608859) | PHAGE_Entero_Arya_NC_031048: hypothetical protein; I35_RS12025; phage(gi100022) | 3.00E-09 |
| 22 | complement(2608957..2609460) | PHAGE_Escher_vB_EcoM_ECO1230_10_NC_027995: putative phage tail protein; I35_RS12030; phage(gi937533291) | 2.80E-45 |
| 23 | complement(2609471..2610640) | PHAGE_Vibrio_vB_VpaM_MAR_NC_019722: tail sheath protein; I35_RS12035; phage(gi428782746) | 4.88E-96 |
| 24 | complement(2610725..2611504) | PHAGE_Ralsto_RSJ2_NC_028988: putative tail fiber assembly potein homolog; I35_RS12040; phage(gi971763581) | 3.74E-59 |
| 25 | complement(2611520..2613538) | PHAGE_Burkho_KL3_NC_015266: gp23; I35_RS12045; phage(gi327198068) | 2.86E-169 |
| 26 | complement(2613526..2614104) | PHAGE_Burkho_phiE255_NC_009237: gp25, tail fiber; I35_RS12050; phage(gi134288819) | 2.25E-36 |
| 27 | complement(2614094..2614990) | PHAGE_Entero_Arya_NC_031048: hypothetical protein; I35_RS12055; phage(gi100013) | 3.00E-58 |
| 28 | complement(2614987..2615322) | PHAGE_Entero_Arya_NC_031048: RecT family recombinase; I35_RS12060; phage(gi100012) | 1.10E-18 |
| 29 | complement(2615322..2615522) | hypothetical protein; I35_RS12065 | 0 |
| 30 | complement(2615582..2616262) | PHAGE_Burkho_ST79_NC_021343: phage baseplate assembly protein V; I35_RS12070; phage(gi509141639) | 2.61E-35 |
| 31 | complement(2616266..2616790) | PHAGE_Entero_Arya_NC_031048: hypothetical protein; I35_RS12075; phage(gi100010) | 1.46E-12 |
| 32 | complement(2616780..2617310) | PHAGE_Halomo_phiHAP_1_NC_010342: hypothetical protein; I35_RS12080; phage(gi167832352) | 4.10E-23 |
| 33 | complement(2617313..2617600) | hypothetical protein; I35_RS12085 | 0 |
| 34 | complement(2617602..2618597) | PHAGE_Escher_JH2_NC_029023: major capsid protein; I35_RS12090; phage(gi971766570) | 1.71E-32 |
| 35 | complement(2618671..2619015) | PHAGE_Gordon_Yvonnetastic_NC_031230: hypothetical protein; I35_RS12095; phage(gi100027) | 3.86E-06 |
| 36 | complement(2619046..2620113) | PHAGE_Pseudo_MD8_NC_031091: putative Cro/Cl-type repressor; I35_RS12100; phage(gi100005) | 1.34E-43 |
| 37 | complement(2620110..2621603) | PHAGE_Vibrio_vB_VpaM_MAR_NC_019722: portal protein; I35_RS12105; phage(gi428782728) | 1.93E-89 |
| 38 | complement(2621600..2621806) | PHAGE_Stenot_S1_NC_011589: putative peptidase b; I35_RS12110; phage(gi213163904) | 1.04E-05 |
| 39 | complement(2621820..2623931) | PHAGE_Entero_Arya_NC_031048: hypothetical protein; I35_RS12115; phage(gi100002) | 6.08E-74 |
| 40 | 2624056..2624283 | hypothetical protein; I35_RS12120 | 0 |
| 41 | complement(2624428..2624622) | hypothetical protein; I35_RS12125 | 0 |
| 42 | complement(2624870..2625643) | hypothetical protein; I35_RS12130 | 0 |
| 43 | complement(2625864..2628371) | PHAGE_Staphy_tp310_2_NC_009762: hypothetical protein; I35_RS12135; phage(gi156603988) | 1.94E-55 |

**Supplementary Table 3.** Bacterial strains used in this study.

| **Strain** | **Description** | **Reference** |
| --- | --- | --- |
| *Burkholderia cenocepacia* H111 | Clinical isolate from CF patient | 1 |
| *Burkholderia cenocepacia* H111 *Δlys1,lys2* | Double mutant strain in genes I35_RS11995 and I35_RS00535 | This study |
| *Burkholderia cenocepacia* H111 *Δlys1,lys3* | Double mutant strain in genes I35_RS11995 and I35_RS07665 | This study |
| *Burkholderia cenocepacia* H111 *Δlys2,lys3* | Double mutant strain in genes I35_RS00535 and I35_RS07665 | This study |
| *Burkholderia cenocepacia* H111 *Δlys1,lys2,lys3* | Triple mutant strain in genes I35_RS11995, I35_RS00535 and I35_RS07665 | This study |
| *Burkholderia cenocepacia* H111 pPROBE-*Pr_recA* | H111 wildtype strain with *recA* promoter fused to GFP into pPROBE plasmid | This study |
| *Burkholderia cenocepacia* H111 *Δlys1,lys2,lys3* + pJN105 | Triple endolysin mutant with the pJN105 plasmid, which contains an arabinose-inducible promoter | This study |
| *Burkholderia cenocepacia* H111 *Δlys1,lys2,lys3* + pJN105*hol_lys* | Triple endolysin mutant with the PA0614 (*hol*) and PA0629 (*lys*) genes cloned into the pJN150 plasmid | This study |

**Supplementary Table 4.** Plasmids used in this study.

| **Plasmid** | **Description** | **Reference** |
| --- | --- | --- |
| pGPI-SceI | Suicide plasmid with oriR6K, mob^+^, I-SceI restriction site; Tp^R^ | 2 |
| pGPI-SceI-*lys1* | pGPI-SceI plasmid with fused regions flanking *lys1* gene | This study |
| pGPI-SceI-*lys2* | pGPI-SceI plasmid with fused regions flanking *lys2* gene | This study |
| pGPI-SceI-*lys3* | pGPI-SceI plasmid with fused regions flanking *lys3* gene | This study |
| pDAI-SceI | pDA17 plasmid carrying the I-SceI nuclease gene; Gm^R^ | 2 |
| pRK2013 | Helper plasmid; RK2 derivative, mob^+^ tra^+^ ori ColE1; Km^R^ | 3 |
| pPROBE-NT | Plasmid carrying promoter-less GFP | 4 |
| pJN105 | *araC*-PBAD cassette cloned in pBBR1MCS-5, Gm^R^ | 5 |
| pJN105*hol_lys* | PA0614 (*hol*) and PA0629 (*lys*) genes cloned into the pJN150 plasmid | This study |

**Supplementary Table 5.** Primers used in this study.

| **Primer** | **Sequence** | **Description** | **Size (bp)** | **Reference** |
| --- | --- | --- | --- | --- |
| *lys1*_fw_up | GAAGGTCGGGCATAAGAAGAAGTCG | To amplify the region upstream *lys1* gene | 610 | This study |
| *lys1*_rv_up | GTCGATCTTCGCAGTTACGTTCATTGC |  |  |  |
| *lys1*_fw_down | CCGGAGAACGAAAAGTTCGAGTACGGC | To amplify the region downstream *lys1* gene | 572 | This study |
| *lys1*_rv_down | CGACCCGAAGAAATACTCCTTCGAGC |  |  |  |
| *lys2*_fw_up | GACCATGAGAGCCATCTTCACTG | To amplify the region upstream *lys2* gene | 984 | This study |
| *lys2*_rv_up | TACAAGCTTCGTCATTCGCTCCTCAGGAG |  |  |  |
| *lys2*_fw_down | TAGAAGCTTCGAACCTGTACGACGTGAAGC | To amplify the region downstream *lys2* gene | 970 | This study |
| *lys2*_rv_down | AATCTAGACCGACCTCGAAGGTGATGC |  |  |  |
| *lys3*_fw_up | CCGACGTCTGCCTGATCAATC | To amplify the region upstream *lys3* gene | 971 | This study |
| *lys3*_rv_up | TACAAGCTTTTCGGTACGTTAGCCATCGAGC |  |  |  |
| *lys3*_fw_down | TACAAGCTTCTGCGAGCGTGGCCTATG | To amplify the region downstream *lys3* gene | 943 | This study |
| *lys3*_rv_down | CATATGTCTAGAACGTCGGCAAGCAGATCG |  |  |  |
| P_*recA*_fw_HindIII | AAAAAAAAGCTTAGGATCAGCAGGTCGAAAGT | To amplify *recA* promoter | 335 | This study |
| P_*recA*_rv_BamHI | AAAAAAGGATCCGAATCGTCCTTTGCTATGAT |  |  |  |
| PA0614_F | CTGAATTCCCAGGGAGGCACTCGTGAAGCACC | To amplify the PA0614 gene of *P. aeruginosa* PAO1 | 468 | This study |
| PA0614H_R | CCGGATCCTCAGTGGTGGTGGTGGTGGTGATGCGGGCCACGGTCGTCCG |  |  |  |
| PA0629_F | CTGGATCCCTGAAACCCATCGGAGTGCAGGAGGATCG | To amplify the PA0629 gene of *P. aeruginosa* PAO1 | 648 | This study |
| PA0629H_R | CCTCTAGATCAGTGGTGGTGGTGGTGGTGTGACAGCACCGCCCTGGC |  |  | 6 |
| pARA | CTGACGCTTTTTATCGCAACTC | To amplify the insert within the pJN105 plasmid | - | This study |
| M13F | GTAAAACGACGGCCAGT |  |  |  |

**
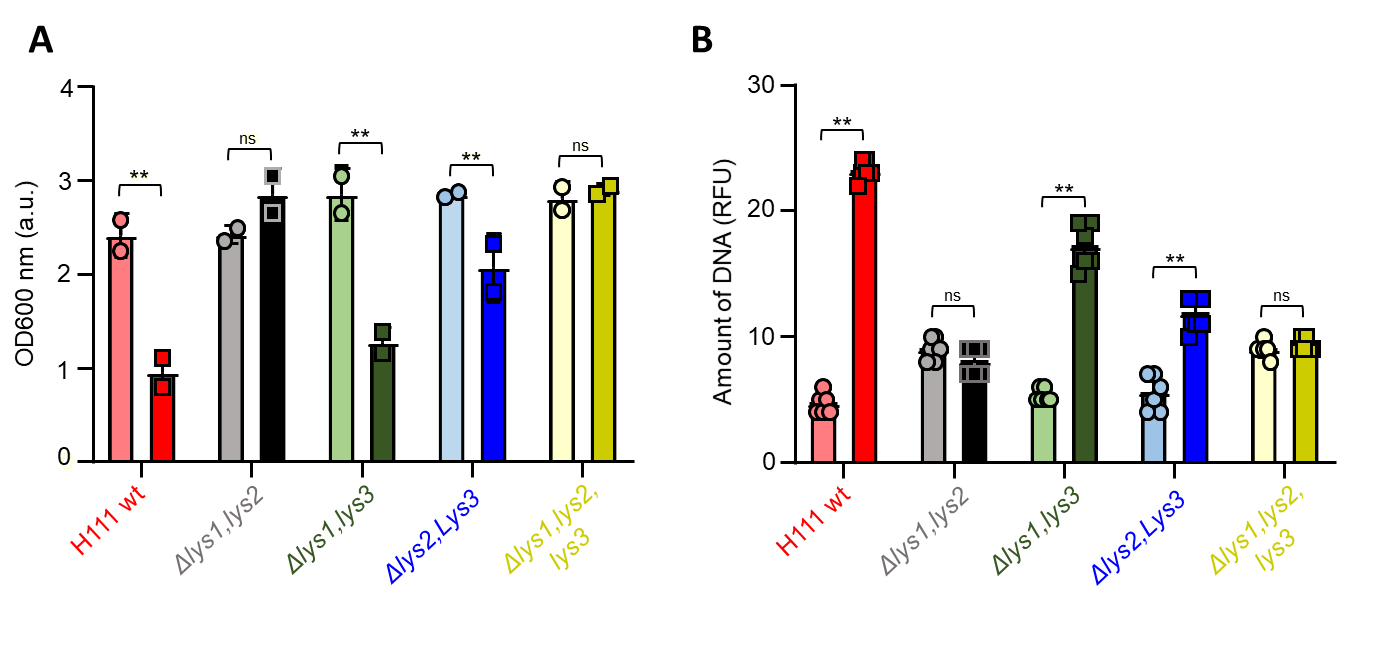
**

**Supplementary Figure 1**. CPFLX-induced explosive cell lysis depends on the endolysins Lys1 and Lys2. Cultures of the H111 wild-type strain and various endolysin mutants were grown for 4 h prior to the addition of 300 ng mL^-1^ CPFLX (right, squares). Control cultures were untreated (left, circles). Samples were taken after 4 h of growth under inducing conditions. (A) OD600; (B) amount of eDNA in cell-free culture supernatants as determined by staining with SYTOX.


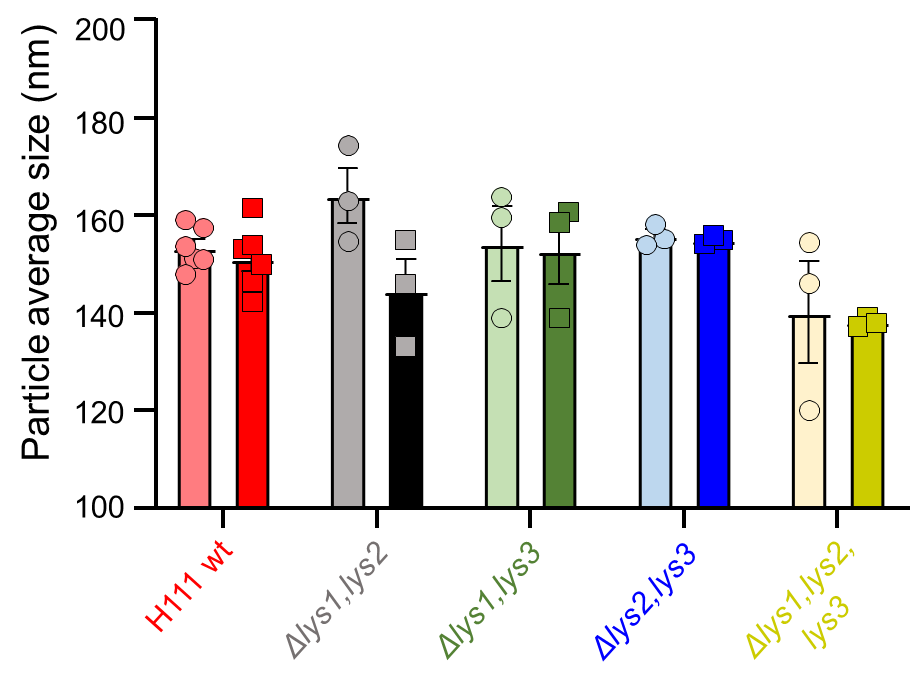


**Supplementary Figure 2.** Particle average size in samples of cell-free supernatant of the H111 wildtype and its derived endolysin mutant strains without treatment (left, circles) and MMC treated (right, squares). Error bars represent the standard error of the mean (SEM). Charts show data obtained from three independent experiments. MMC treatment 200 ng mL^-1^.

**Supplementary Figure 3.** Bacterial surface coverage (percentage) (A) and red fluorescence intensity (B) measured on the horizontal surface of the channel colonized by *B. cenocepacia* H111 wt (red), H111 *Δlys1,lys2* (black), H111 *ΔLys1,lys3* (green), H111 *Δlys2,lys3* (blue) and H111 *Δlys1,lys2,lys3* (yellow) cells, measured after 15 h of a continuous flow of a diluted bacterial suspension at *U* = 2 mm/s, containing ciprofloxacin at concentrations of 0 ng mL^-1^ (left) and 300 ng mL^-1^ (right). Each data point reports the results obtained on 200 x 675 µm areas located in same microfluidic channels where streamers are grown in Fig.3. Three positions are taken in six different channels (2 technical replicates per experiment; 3 biological replicates). Error bars represent the standard error of the mean (SEM).


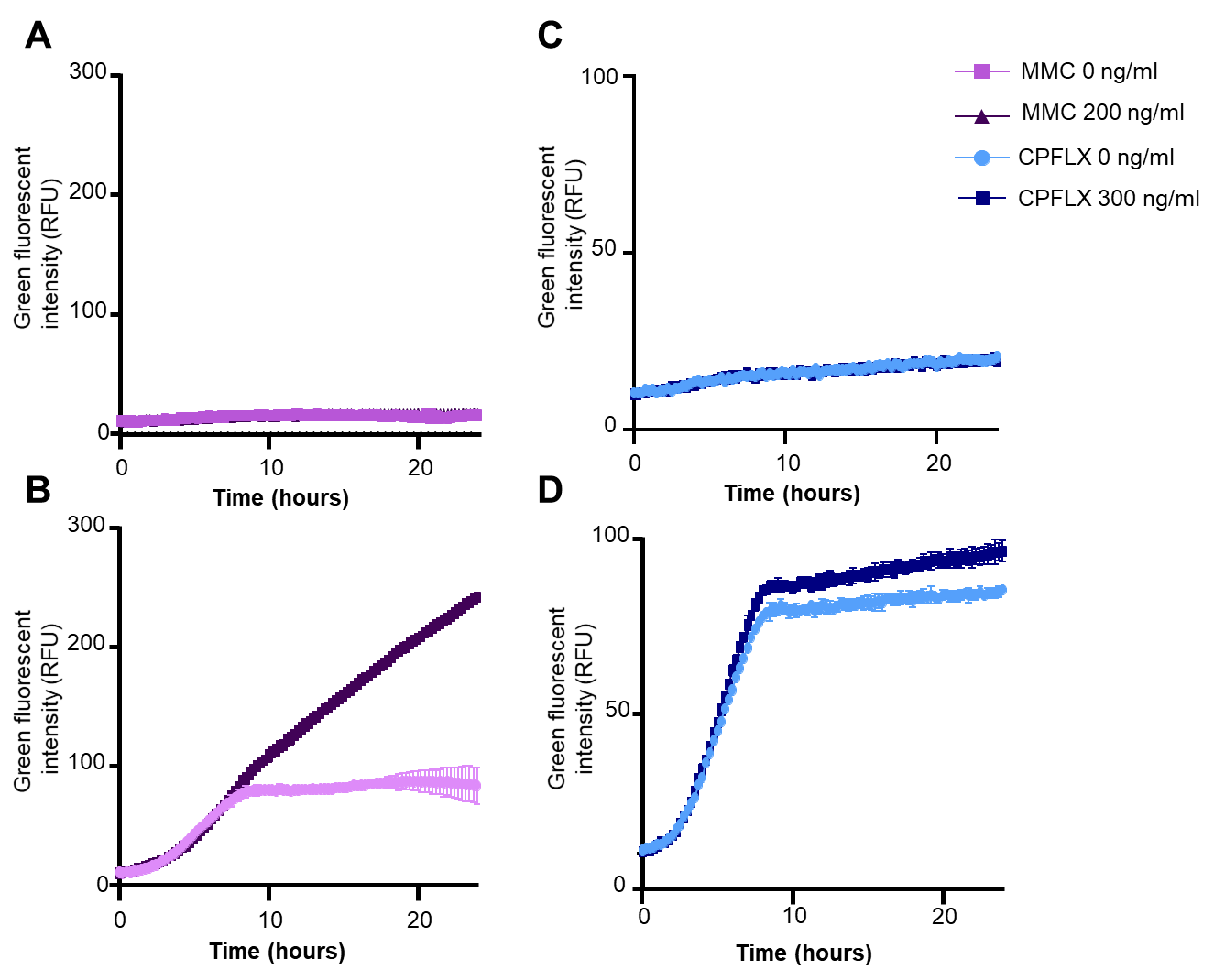


**Supplementary Figure 4.** Functional validation of an SOS response reporter. The *recA* promoter was fused to GFP in the pPROBE plasmid and introduced into H111 wildtype. The empty pPROBE plasmid was used as a negative control. (A, C) empty pPROBE plasmid; (B, D) *recA* promoter fused to GFP in the pPROBE plasmid. MMC treatment 200 ng mL^-1^; CPFLX treatment 300 ng mL^-1^.


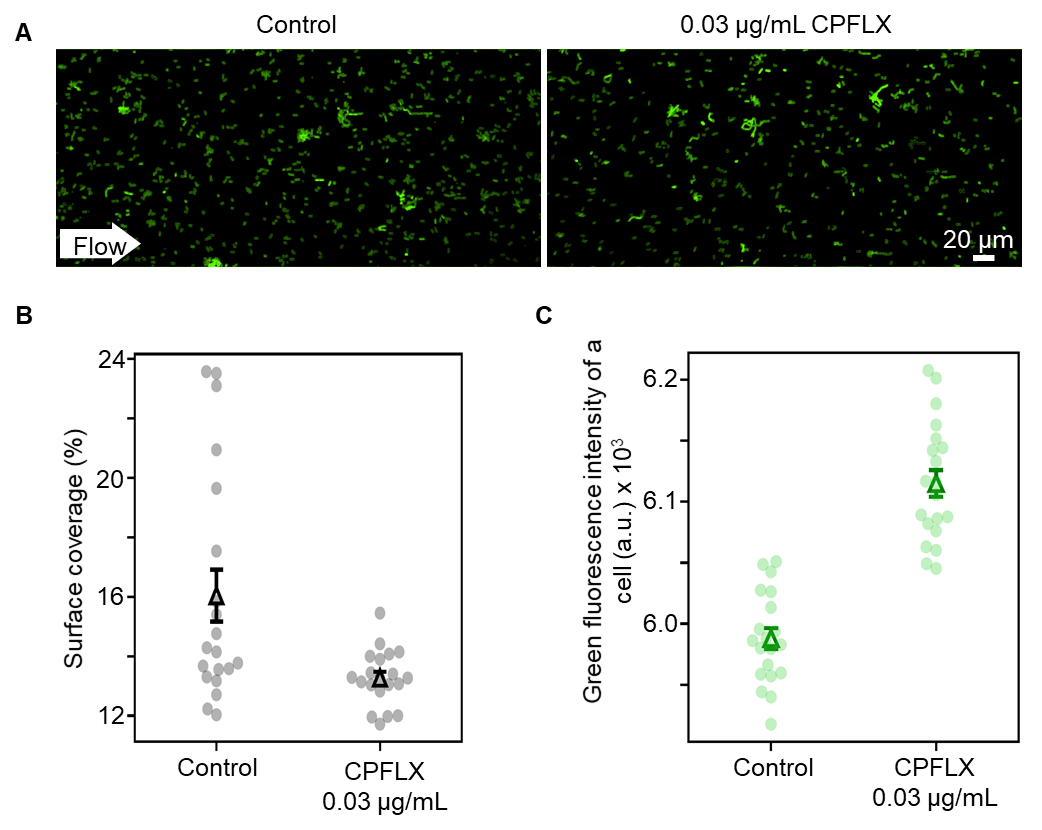


**Supplementary Figure 5.** (A) Representative green fluorescence of *B. cenocepacia* H111 *Pr_recA* cells attached to the glass surface of the microfluidic channel after 15 h of a continuous flow of a dilute bacterial suspension at *U*= 2 mm/s, containing CPFLX at concentrations of 0 ng mL^-1^ (left) and 300 ng mL^-1^ (right); (B) Bacterial surface coverage (percentage) and (C) average green fluorescence intensity of a single bacterial cell measured in the same conditions as in panel A. Each data point reports the results obtained on 200 x 675 µm areas located in same microfluidic channels where streamers are grown in Fig.3. Three positions are taken in for different channels (2 technical replicates per experiment; 2 biological replicates). Error bars represent the standard error of the mean (SEM).

**Supplementary Figure 6.** (A) Quantification of proteins in cell-free supernatants of the H111 wildtype and the various endolysin mutants using the Pierce^TM^ BCA Protein Assay Kit (Thermo Fisher); (B) Quantification of lipids in cell-free supernatants of the H111 wildtype and the various endolysin mutants using the FM^TM^ 1-43 dye (Invitrogen).

**References:**

1. Carlier, A. *et al.* Genome Sequence of *Burkholderia cenocepacia* H111, a cystic fibrosis airway isolate. *Genome Announc*. **2**(2): e00298-14. doi: 10.1128/genomeA.00298-14 (2014).
2. Flannagan, R.S., Linn, T. & Valvano, M.A. A system for the construction of targeted unmarked gen deletions in the genus *Burkholderia*. *Environ Microbiol.* 10:1652-60 (2008).
3. Figurski, D. H. & Helinski, D. R. Replication of an origin-containing derivative of plasmid RK2 dependent on a plasmid function provided in *trans*. *Proc. Natl Acad. Sci*. USA **76**(4), 1648–1652 (1979).
4. Miller, W. G., Leveau, J. H. & Lindow, S. E. Improved gfp and inaZ broad-host-range promoter-probe vectors. *Mol. Plant Microbe Interact*. **13**(11), 1243–1250 (2000).
5. Newman, J. R. & Fuqua, C. Broad-host-range expression vectors that carry the L arabinose- inducible *Escherichia coli* araBAD promoter and the araC regulator. *Gene* **227**(2), 197–203 (1999).
6. Turnbull, L., Toyofuku, M., Hynen, A.L., Kurosawa, M., Pessi, G. *et al.* Explosive cell lysis as a mechanism for the biogenesis of bacterial membrane vesicles and biofilms. *Nat Commun*. **7**, 11220; 10.1038/ncomms11220 (2016).
