# Supplementary material for: Genotoxic stress stimulates eDNA release via explosive cell lysis and thereby promotes streamer formation of Burkholderia cenocepacia H111 cultured in a microfluidic device
Source: NPJ Biofilms Microbiomes. 2023 Dec 9;9:96. doi: 10.1038/s41522-023-00464-7 (PMC10710452; doi:10.1038/s41522-023-00464-7)
Supplement: Supplementary file 4 — SupplementaryVideo [file 41522_2023_464_MOESM4_ESM.docx]

**Video 1**. **MMC induces explosive cell lysis in *B. cenocepacia* H111.** Cells were grown in the presence of MMC (200 ng ml^-1^). Bacterial cells (green); DNA (magenta).
